# The cardiac-restricted protein ADP-ribosylhydrolase-like 1 is essential for heart chamber outgrowth and acts on muscle actin filament assembly

**DOI:** 10.1016/j.ydbio.2016.05.006

**Published:** 2016-08-15

**Authors:** Stuart J. Smith, Norma Towers, José W. Saldanha, Catherine A. Shang, S. Radma Mahmood, William R. Taylor, Timothy J. Mohun

**Affiliations:** a Heart Formation in Vertebrates Laboratory, The Francis Crick Institute – Mill Hill Laboratory, The Ridgeway, Mill Hill, London NW7 1AA, UK; b Mathematical Biology Laboratory, The Francis Crick Institute – Mill Hill Laboratory, The Ridgeway, Mill Hill, London NW7 1AA, UK; c Experimental Histopathology, The Francis Crick Institute – Mill Hill Laboratory, The Ridgeway, Mill Hill, London NW7 1AA, UK

**Keywords:** Adprhl1, Heart, Ventricle, Myofibril, Morpholino, Transgenic

## Abstract

Adprhl1, a member of the ADP-ribosylhydrolase protein family, is expressed exclusively in the developing heart of all vertebrates. In the amphibian *Xenopus laevis*, distribution of its mRNA is biased towards actively growing chamber myocardium. Morpholino oligonucleotide-mediated knockdown of all Adprhl1 variants inhibits striated myofibril assembly and prevents outgrowth of the ventricle. The resulting ventricles retain normal electrical conduction and express markers of chamber muscle differentiation but are functionally inert. Using a cardiac-specific Gal4 binary expression system, we show that the abundance of Adprhl1 protein in tadpole hearts is tightly controlled through a negative regulatory mechanism targeting the 5′-coding sequence of *Xenopus adprhl1*. Over-expression of full length (40 kDa) Adprhl1 variants modified to escape such repression, also disrupts cardiac myofibrillogenesis. Disarrayed myofibrils persist that show extensive branching, with sarcomere division occurring at the actin-Z-disc boundary. Ultimately, Adprhl1-positive cells contain thin actin threads, connected to numerous circular branch points. Recombinant Adprhl1 can localize to stripes adjacent to the Z-disc, suggesting a direct role for Adprhl1 in modifying Z-disc and actin dynamics as heart chambers grow. Modelling the structure of Adprhl1 suggests this cardiac-specific protein is a pseudoenzyme, lacking key residues necessary for ADP-ribosylhydrolase catalytic activity.

## Highlights

• Adprhl1 is expressed exclusively in the heart of all vertebrates.
• Morpholino knockdown of Adprhl1 prevents outgrowth of the ventricle.
• Elevated 40 kDa Adprhl1 produces disarrayed myofibrils that show extensive branching.
• The 5′-coding sequence of *Xenopus adprhl1* influences the synthesis of Adprhl1 protein.
• Two Adprhl1 proteins, 40+23 kDa exist in *Xenopus* embryos and are conserved in mouse.

## 1 Introduction

In all vertebrate embryos, the chambers of the heart arise by transformation of a simple, linear myocardial muscle tube. As cardiac beating commences, this tube subsequently loops with a counter-clockwise, helical rotation and chambers balloon out from regions of the outer curvature. In recent years, detailed descriptions of heart developmental anatomy have been produced (de Boer et al., 2012, Mohun and Weninger, 2011, Xavier-Neto et al., 2012) and transcription factors identified that are important for the correct emergence of the cardiac chambers (reviewed Greulich et al., 2011, McCulley and Black, 2012). Nonetheless, identifying the downstream mechanisms that ensure the precise construction of chamber structures still present a major challenge.

Myofibrillogenesis, the process of assembling the contractile protein machinery within muscle cells of the embryonic heart, occurs concurrently with chamber formation. In fact, myofibril growth is intimately linked to increasing cardiomyocyte cell size and the coordinated changes in cell shape that underpin chamber outgrowth (Lin et al., 2012). In mouse embryos, myofibrils are known to assemble near the surface of chamber cardiomyocytes and initially extend in diverse directions to produce multiple junctions with neighbouring cells. As these myofibrils lengthen, they gradually acquire an increasingly parallel orientation. However, true longitudinal growth, where myofibrils are fully aligned and adjacent cells have the same polarity, only occurs during late stages of chamber maturation. This occurs, for example, within the ventricle compact layer observed beyond E16.5 and after birth (Hirschy et al., 2006). Regional variations in myofibril patterns also exist within the early forming chambers. This variability goes beyond the obvious differences between myofibril orientations in ventricle trabeculae compared to the external free walls (Price et al., 1996). Within the simple heart of zebrafish, distinct myofibril arrangements are found in the ventricle compared to the atrium. Moreover, pattern differences have also been noted between ventricular myofibrils residing at different depths within the same cardiomyocyte (Reischauer et al., 2014). Myofibril assembly measured in living embryos shows regional responses within the ventricle; not all cardiomyocytes grow and deposit myofibrils within a monitored time frame, but those that are hypertrophic will frequently have neighbouring cells that are also growing (Lin et al., 2012). It seems likely that such heterogeneity in myofibrillogenesis will be a major feature of ventricle formation in higher vertebrates, given the added asymmetry that accompanies outgrowth of more complex chambers. Cells must behave asynchronously in order that an asymmetric structure be constructed.

In models that describe the sequence of myofibrillogenesis (Liu et al., 2013, Sparrow and Schock, 2009), rearrangement of the actin cytoskeleton in the cell cortex is a crucial early step. This requires disassembly, as well as filament nucleation and elongation to produce sarcomeric actin filaments with uniform length and polarity. However, such models rarely consider the trajectory of myofibril assembly, an aspect that is critical to understanding how embryonic cardiomyocytes are incorporated into heart chambers; furthermore mechanisms to explain such directionality are simply not known. It is also unclear to what extent myofibrils that have different orientations within a cell are structurally connected to each other during this phase of chamber outgrowth. In this study, we have identified a novel protein, Adprhl1, whose action modifies early cardiac actin dynamics and myofibril directionality.

Adprhl1 is so named because of its sequence similarity to a small group of ADP-ribosylhydrolase enzymes encoded in vertebrate genomes; Adprh (sometimes named ARH1), Adprhl1 (ARH2) and Adprhl2 (ARH3)(pfam03747). Mono-ADP-ribosylation of proteins is a post-translational modification catalysed by ADP-ribosyltransferase enzymes and also many bacterial toxins (reviewed Aktories et al., 2011, Laing et al., 2011). It appears to be a reversible modification, with ADP-ribosylhydrolase (Adprh) a 357 amino acid (in human) cytosolic protein that can cleave the ADP-ribose linkage with arginine (protein) side chains (Moss et al., 1985, Moss et al., 1992, Okazaki et al., 1995). Natural protein targets of Adprh are unclear and while mice lacking *Adprh* are viable, evidence from the knockout indicates that *Adprh* somehow acts as an important tumour suppressor (Kato et al., 2011). Expression of *adprh* in zebrafish embryos is detected transiently within forming somites, suggesting ADP-ribosylation might participate in skeletal muscle development. Study of another member of this homologous gene family, ADP-ribosylhydrolase-like 2 (Adprhl2), which shares 22% amino acid sequence identity with Adprh, has revealed it can act on two distinct classes of substrates. Mammalian Adprhl2 hydrolyzes poly(ADP-ribose) chains to release ADP-ribose monomers (Oka et al., 2006), potentially opposing the activity of the poly(ADP-ribose) polymerases that signal during DNA repair and chromatin remodelling. Adprhl2 can also hydrolyze O-acetylated-ADP-ribose (Kasamatsu et al., 2011, Ono et al., 2006), the reaction by-product of Sir2 protein deacetylases, but does not act on mono-ADP-ribose linked to amino acids.

ADP-ribosylhydrolase-like 1 (Adprhl1) (Oka et al., 2006) is another member of this protein family, the 354 amino acid sequence of human Adprhl1 showing 46% identity to Adprh. Intriguingly, in contrast to the other two proteins, Adprhl1 appears to lack any comparable enzymatic activity (Oka et al., 2006). The strong evolutionary conservation of Adprhl1 sequence extends to frogs, with *Xenopus* Adprhl1 being 75% identical to human Adprhl1 and 47% identical to the *Xenopus* species Adprh. Here, we have studied Adprhl1 function using embryos of the frog, *Xenopus laevis*. Models of the Adprhl1 protein structure explain the lack of enzymatic activity compared to other family members. We have identified expression of its mRNA within actively growing heart chamber myocardium in *Xenopus* embryos. Gene knockdown and over-expression experiments demonstrate Adprhl1 is essential for heart chamber outgrowth, alteration of Adprhl1 expression levels impacting upon myofibril assembly. Elevated Adprhl1 is associated with disarrayed myofibril patterns, contractile filaments with diverging orientations and prominent branches at the actin-Z-disc boundary. Finally, we demonstrate that in normal development, Adprhl1 production is subject to tight regulatory control, mediated through targeting of the 5′-coding sequence of *Xenopus adprhl1*.

## 2 Materials and methods

### 2.1 *Adprhl1* gene and cDNA sequences

*X. laevis adprhl1*: Xenbase XB-GENE-989263. JGI *X. laevis* genome 7.2 scaffold 167628: 4825123-4840241. A potential second locus Xelaev16044576m: Genome 7.2 scaffold 338390: 1306406-1316841. cDNA NCBI MGC:82403, IMAGE:4409193. Additionally, intron 2 genomic isoforms from the outbred animals used in the study were sequenced (Accession GU188989, GU188990) for morpholino design. Human *ADPRHL1*: NCBI Gene ID 113622. Full length cDNA NM_138430. Shorter coding transcript NM_199162. The full length human *ADPRHL1* cDNA used IMAGE:5299214, but it contained a sequence variation giving a Pro^248^His substitution. Expression constructs therefore exchanged a *PstI-SacI* fragment from the shorter clone, IMAGE:3838063, to maintain the evolutionarily conserved Pro^248^ residue. Mouse *Adprhl1*: NCBI Gene ID 234072. cDNA NM_172750. Wholemount *in situ* hybridization probes comprised all of the coding sequence, or from 277 to 1065bp for comparison of transgenic line activity.

### 2.2 *Adprhl1* morpholino sequences

Morpholino oligonucleotides (Gene-Tools) were designed to interfere with *X. laevis adprhl1* RNA splicing.

Adprhl1-e2i2MO 5′-AGGCTCAGCATCTTACAAACCTTTT-3′.
Adprhl1-i2e3MO 5′-ACCTAAGAAACAACTAGAGTCACTG-3′.
Adprhl1-e2i2MOMis 5′-AAGCTAAGCATATTAAAAACATTTT-3′.
Adprhl1-i2e3MOMis 5′-AACTAAAAAACAAATAGAATCAATG-3′.
Adprhl1-ControlMOMis 5′-GATAGTTATAAGTAATTCTACCAAT-3′.

### 2.3 Morpholino injection into *Xenopus* embryos

A 32 ng dose of the splice interfering *adprhl1* morpholino oligonucleotides was injected at the four-cell stage, either 2 nl of a 8 ng/nl stock was injected into both dorsal blastomeres (D-2/4) or as a control into both ventral blastomeres (V-2/4). Injection into dorsal blastomeres distributes morpholino into cells of the forming heart whereas control ventral injection distributes morpholino into posterior-ventral portions of the tadpole. For rescue of the *adprhl1* morpholino heart defect, a 24 ng mass of morpholino was used, combined with 240–600 pg *adprhl1* RNA. Standard procedures were employed (Sive et al., 2000).

### 2.4 *Xenopus* embryo analysis

*Adprhl1* RT-PCR was performed on individual stage 30 embryos, or 50 dissected stage 42 hearts, with RNA isolated in 150 μl TRIzol reagent, DNA-free (Ambion) and SuperScriptIII Reverse Transcriptase (Invitrogen). Equivalent control reactions that did not have reverse transcriptase added were used for all embryo RNAs. Standard procedures were used for wholemount RNA *in situ* hybridization (Harland, 1991, Sive et al., 2000) with antisense probes for cardiac markers (Smith et al., 2005, Smith and Mohun, 2011) and for *Xenopus ami* (adipsin, Image:6933579).

### 2.5 Calcium imaging using R-GECO1

Robert Campbell kindly donated the plasmid, CMV-R-GECO1 (Addgene:32444) (Zhao et al., 2011). A pCS2-R-GECO1 plasmid was constructed for transcription of sense RNA. Embryos were co-injected with 20 ng Adprhl1-e2i2MO and 800 pg R-GECO1 RNA into D-2/4 blastomeres. A lower 20 ng mass of morpholino was used in this experiment to accommodate co-injection of the RNA into embryos. Thus a reduced proportion of tadpoles developed the complete phenotype associated with the Adprhl1-e2i2MO (20 out of 42 tadpoles injected, 48%). For time-lapse movies, greyscale (8-bit) images were captured using a Q-Imaging camera and Image-Pro software at 20 frames per second for 10 s, then false coloured using the GEM LUT within Image J software (Schneider et al., 2012).

### 2.6 Adprhl1 antibody and immunocytochemistry of *Xenopus* hearts

The peptide sequence used to raise a polyclonal rabbit antibody against Adprhl1 was: ^248^DNYDAEERDKTYKKWSSE^265^ (Cambridge Research Biochemicals). The sequence derives from mouse ADPRHL1, yet the antibody is also active against the *Xenopus* and human orthologs (aa residues 249-266). Other antibodies used were: Myosin A4.1025 (DSHB), Actin 20-33 A5060 (Sigma), Sarcomeric α-actinin EA-53 (abcam), FLAG M2 F1804 (Sigma), HA 3F10 (Roche), NKX2-5H-114 (Santa Cruz). Fluorescent dye-conjugated secondary antibodies (Jackson ImmunoResearch) and Alexa Fluor568-conjugated phalloidin (Invitrogen) were used to visualize myofibril components. Immunocytochemistry was performed on whole tadpoles and subsequently the hearts were dissected, mounted in 12 μl CyGEL Sustain (biostatus) and viewed using a Zeiss LSM5-Pascal confocal or a Zeiss Axioimager microscope. The confocal images of whole hearts captured 2 µm deep optical sections. Images of myofibrils were 1 µm optical sections, at a depth 1–2 µm below the outer (apical) myocardial surface. Cell number counts of the heart surface at stage 33 utilised cortical filament staining to discriminate cell boundaries while stage 37 and 40 ventricles used cell nuclei counts with Image J.

### 2.7 Recombinant Adprhl1 proteins

DNA constructs for expression of recombinant Adprhl1 proteins utilised solely the coding cDNAs with no endogenous untranslated sequences present. Hybrid human-*Xenopus* Adprhl1 protein constructs were produced by utilizing restriction sites that were present in the cDNA of one species and engineering the same site into the second species by PCR. Sites used were *Msc1* (at 157–162 bp in *Xenopus adprhl1* cds), *HinDIII* (at 277–282 bp in human *ADPRHL1* cds) and *Sac1* (at 789–794 bp in both species cds). GeneArt Strings (Invitrogen) synthesized DNA fragments were also employed in order to achieve discrete sequence changes in some Adprhl1 constructs (the small peptide switches and the silent nucleotide changes). When designing artificial construct sequences, the *Xenopus laevis* codon usage table (Musto et al., 2001) was consulted to avoid rare occurring codons.

### 2.8 Binary system for cardiac Adprhl1 expression using myl7:Gal4/UAS:responder transgenes

Transgenes were incorporated into *Xenopus* embryos by the sperm nuclear injection transgenesis method (Smith et al., 2006). A binary transgene system was adopted for most cardiac over-expression experiments, that still incorporated and allowed analysis of new *adprhl1* containing transgenes in the founder generation (S. Fig. 12). A *Tg[myl7:Gal4, γCrys:eCFP]* driver transgenic frog line was established and a sperm nucleus preparation was isolated from a f1 generation male. New responder transgenes, such as *Tg[UAS:Xenopus adprhl1, γCrys:DsRed1]* , were then added to the driver line sperm in a standard transgenesis procedure. After injection to fertilize eggs, healthy embryos were selected at stage 28, before transgenes became active. At stage 40, tadpoles containing both transgenes were identified by their cyan and red-fluorescent eyes and they were analyzed for Adprhl1 protein production at stage 44. The transgenes used in the study are listed in S. Fig. 12, while their names are abbreviated when described in the Results section. Stable lines for key responder transgenes were subsequently established (S. Fig. 12).

### 2.9 Models of Adprhl1 structure

BLAST searching the Protein Data Bank (PDB) sequences identified the template structure for model building, the crystal structure of human ADPRH (PDB ID: 3HFW) (Kernstock et al., 2009), which shares 47% identity with *Xenopus* Adprhl1. Initial alignments were manually refined to avoid indels within elements of secondary structure. Protein structural models of human and *Xenopus* Adprhl1, plus each of the hybrid proteins were built using the program, Modeller (Sali and Blundell, 1993). Modeller uses homology-derived restraints of dihedral angles and distances for main-chain and side-chain atoms. Regions lacking template coordinates were built by an energy function optimization approach based on energy minimization using conjugate gradients and molecular dynamics with simulated annealing. The stereochemical quality of the models was assessed by QMEAN Z-scores (Benkert et al., 2011), which ranged from −2.81 for the *Xenopus* model to −3.33 for the human^1-52^-*Xenopus*^53-354^ hybrid Adprhl1, indicating reliable models. Each of the hybrids superposed well, with only minor deviations in loop regions and side chain conformations compared with the entirely human or *Xenopus* Adprhl1 proteins, suggesting they would fold correctly.

## 3 Results

### 3.1 *Adprhl1* mRNA is expressed during heart chamber outgrowth

**Fig. 1:**
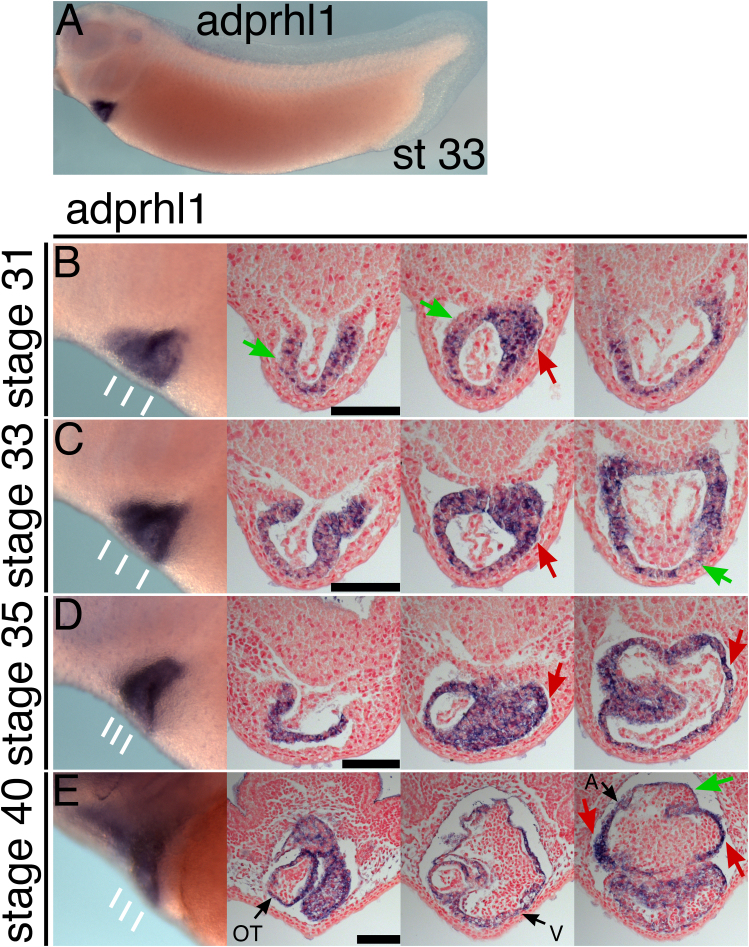
*Myocardial expression of adprhl1 in Xenopus embryos*. Developmental stage series of cardiac *adprhl1* mRNA expression in *X. laevis* embryos. A: Left-lateral view of a stage 33 tadpole with *adprhl1* mRNA detected solely in the forming heart. B–E: *Adprhl1* expression at stage 31 (B), stage 33 (C), stage 35 (D) and stage 40 (E). Left-lateral view of the heart has white lines marking planes of three transverse sections for each embryo. Red arrows indicate myocardium containing high concentration of *adprhl1* mRNA while green arrows indicate areas with lesser expression. Nuclei counterstained red. Scale bars=100 µm. OT, outflow tract; V, ventricle; A, atrium.

In *Xenopus* embryos, the onset of *adprhl1* expression coincides with early myocardial differentiation (Fig. 1). Wholemount *in situ* hybridization detects *adprhl1* mRNA in cardiac tissue from stage 29 and moreover, the greatest concentrations are found in regions of actively growing chamber myocardium. For example, within the central section of the heart tube during looping stages, the left side contains abundant *adprhl1* mRNA (Fig. 1B and C) and will subsequently contribute to the apex and anterior wall of the ventricle (Ramsdell et al., 2006). Moreover at stage 40, both left and right lateral sides marking the growing atrial chambers stain strongly for *adprhl1* (Fig. 1E) whereas the dorsal atrial roof contains less of the message. Cardiac *Adprhl1* expression is conserved in mammals, with *Adprhl1* mRNA detected within embryonic mouse hearts at E11.5 and there is also preliminary evidence its expression depends on the presence of the cardiac transcription factor, *Nkx2-5* (S. Fig. 1).

### 3.2 *Adprhl1* knockdown prevents heart ventricle growth and function

**Fig. 2:**
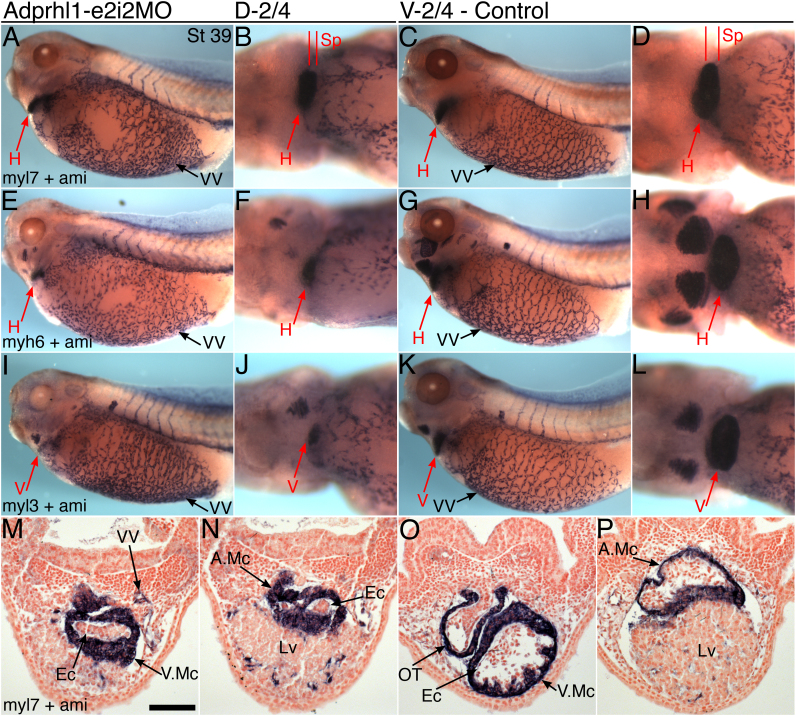
*Adprhl1 morpholino produces small, inert cardiac ventricles*. A, B, E, F, I, J: Stage 39 tadpoles injected with Adprhl1-e2i2MO into D-2/4 blastomeres show detectable expression of *myl7* (*mlc2,* A, B), *myh6* (*mhcα*, E, F) and *myl3* (*mlc1v*, I, J) in their smaller cardiac ventricles. C, D, G, H, K, L: Control stage 39 tadpoles that received the same morpholino into V-2/4 blastomeres. Left lateral views (A, C, E, G, I, K) and ventral views of heart (B, D, F, H, J, L), anterior to left. Vascular expression of *ami* (adipsin) is additionally shown to demonstrate that lateral vitelline vasculature, posterior cardinal vein and intersomitic vessels form in the absence of a functional heart beat (bare VV patches in A, E due to damage to tadpole during procedure). M, N: Representative transverse heart sections of the D-2/4-morpholino tadpole presented (A, B). O, P: Heart sections through the control tadpole (C, D). Section planes (Sp) indicated (B, D). Scale bar=100 µm. H, heart; V, ventricle; A, atrium; Mc, myocardium; Ec, endocardium; OT, outflow tract; VV, vitelline vessel; Lv, liver.

**Fig. 8:**
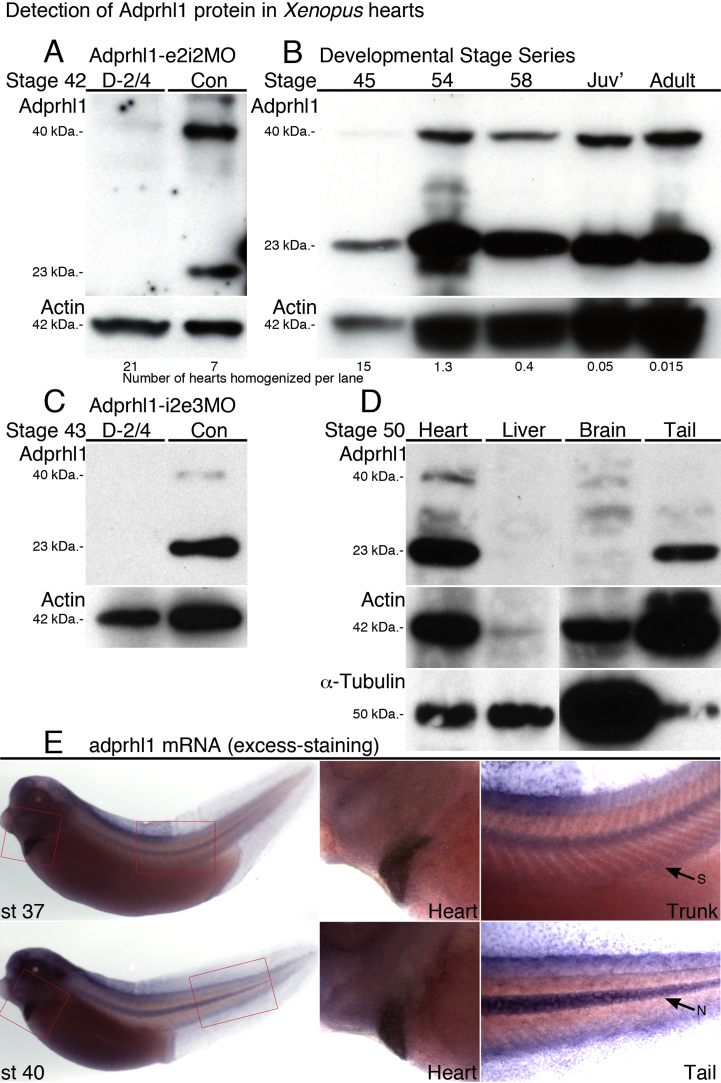
*Detectionof Adprhl1 protein in Xenopus hearts and absence in morpholino injected embryos.* A: Western blot detection of Adprhl1 protein in stage 42 hearts and its absence after injection of Adprhl1-e2i2MO into D-2/4 blastomeres. 21 of the small, morphant hearts were harvested compared to 7 controls, while detection of actin was used to normalize the samples. Two Adprhl1 protein bands, 40 and 23 kDa, were identified in control hearts, only. B: Developmental stage series showing the reversed, relative abundance of the two Adprhl1 protein species in older hearts. C: Injection of the second, Adprhl1-i2e3MO produced a similar loss of Adprhl1 protein. D: Adprhl1 detection within embryonic tissues showed the 40 kDa protein was heart-specific whereas the 23 kDa species was also found in dissected tail pieces. E: Tadpoles excessively stained for *adprhl1* mRNA gave a signal in somite (S) and notochord (N) tissue, suggesting a possible tail source for the 23 kDa protein (29 h substrate incubation instead of the typical 2–4 h).

We examined the effect of knocking down *Xenopus adprhl1* activity using morpholino oligonucleotides (MO) that target RNA splicing, to inhibit production of a functional mRNA. Most experiments used the Adprhl1-e2i2MO morpholino but comparable results were also obtained using an Adprhl1-i2e3MO morpholino (S. Figs. 2 and 8C). Tadpole hearts were analyzed through a range of developmental stages, from stage 33 at the onset of looping of the tubular heart, through to stage 42, when cardiac chamber formation has occurred (and the tadpole has increased transparency). *Adprhl1* knockdown produces a consistent, specific and profound defect in embryonic cardiogenesis. Hearts that received the *adprhl1* morpholinos form with small ventricles that do not beat, as shown at stage 40–41 (S. Fig. 3, S. Movie 1). Beyond stage 41, oedemas develop in tadpoles with impaired cardiac function (Smith et al., 2007) and this also proved to be the case for loss of *adprhl1* (S. Fig. 3).

Co-injection of morpholino along with RNA encoding the red fluorescent calcium indicator protein, R-GECO1, allowed qualitative measurement of cytosolic calcium concentration changes within the ventricular cardiomyocytes. Inert hearts caused by loss of *adprhl1* still exhibited strong calcium waves that propagated across the ventricle, observed at stage 42 (S. Movie 2, Movie 3). In total, 22 morphant hearts were studied with R-GECO1 and none showed a block in electrical conduction (S. Fig. 4), indicating that the failure in heart function induced by *adprhl1* knockdown was not due to loss of electrical impulse generation or propagation.

### 3.3 *Adprhl1* knockdown does not affect cardiac gene expression, nor disrupt early morphology

Expression of cardiac muscle marker mRNAs, such as *myl7* (*mlc2*), *myh6* (*mhcα*) and ventricular *myl3* (*mlc1v*) was not affected by *adprhl1* knockdown (Fig. 2) but histological analysis revealed a profound defect in endocardium maturation in the morphants at stage 39, with endocardial cells having a rounded morphology and no clear lumen forming within the hearts (Fig. 2M, N).

*Xenopus* hearts commence peristaltic beating earlier, at stage 33–34. Analysis at this stage (S. Fig. 5) showed that in the central section, myocardial layers were comparable in both morphant and control heart tubes. However, while the endocardial cells were beginning to organize and form a lumen in controls, no such structure could be identified in the morphant hearts (S. Fig. 5F, J). Additionally, in the outflow tract portion, an open myocardial trough extended anteriorly in control hearts, but was absent in the inert, morphant hearts (S. Fig. 5E, I). Similar effects had been noted previously in a study of a *Xenopus myh6* mutant (Abu-Daya et al., 2009), suggesting that both abnormalities may be secondary consequences of the absence of myocardial beating.

### 3.4 *Adprhl1* knockdown disrupts myofibril assembly in the forming ventricle

Given the profound functional defect after *adprhl1* knockdown, we examined the assembly of myofibrils within the forming ventricle. Three developmental stages were assayed; at stage 33 as looping commences, stage 37, as ventricle outgrowth would normally be occurring and at stage 40, when chamber myofibrillogenesis should be well established.

In control stage 33 heart tubes, actin filaments stained with phalloidin had a cortical localization, while striated patterns were already evident for muscle myosin fibres (Fig. 3C, D, also S. Fig. 6E–H) (S. Fig. 6 includes extra data for Fig. 3 hearts). While most cardiomyocytes had an uneven, rounded shape, some early cell movements driving looping morphogenesis had occurred. Notably, near the apex of the presumptive ventricle, a group of cells on the left side had elongated to form a rosette structure (Fig. 3C, D-arrowheads, Fig. 3J, S. Fig. 6E, F). However, in heart tubes that received the *adprhl1* morpholino, cortical actin filaments revealed that none of the apex cardiomyocytes had elongated (Fig. 3A, S. Fig. 6A, C). Moreover, muscle myosin production within the same region was delayed (Fig. 3B, S. Fig. 6B, D).

By stage 37, control ventricles contained a characteristic arrangement of myofibrils, with both actin and myosin now organized into striated filaments (S. Fig. 6M–P). Around the apex, larger myofibrils of the ventricle wall (S. Fig. 6N–P-coloured arrowheads) extended perpendicularly to axes (Fig. 3K–N) running from base to apex (perpendicular to circumference-axes imagined from inner to outer curvature). Myofibrils closer to the base of the ventricle (the inner curvature) aligned parallel to the axes. Knockdown of *adprhl1* severely disrupted this myofibril assembly. Actin filaments remained predominantly cortical, within rounded cells (S. Fig. 6I–L). Myosin had accumulated by stage 37 but the resulting myofibrils in the ventricle appeared short and disarrayed, with no consistent orientation (S. Fig. 6K, L-white arrowheads). In control hearts at stage 40, the number and width of myofibrils in the ventricle wall was increased while their principal orientation was maintained (Fig. 3G, H, S. Fig. 6U–X). Additional structural complexity developed towards the lumenal surface, where cardiomyocytes of the forming trabeculae elongated parallel to the base to apex axes (Fig. 3K–N). In *adprhl1* morphants, short myofibrils did ultimately accumulate but there was no recovery of their alignment within the ventricle (Fig. 3E, F, S. Fig. 6Q–T).

At the outset of cardiac morphogenesis, equivalent numbers of cardiomyocytes were present within morphant heart tubes compared to controls (Fig. 3I). However, as ventricle outgrowth proceeded, and failed in morphants, progressively fewer cells could be counted on the ventricle surface (Fig. 3I). This could result from reduced incorporation of cells from the progenitor pool into an inert heart but would also occur because the obvious disorder had caused cells to be overlaid (see Fig. 2M). Other aspects of cell structure were unaffected by the *adprhl1* morpholino. Co-injecting RNA for membrane-tagged (lyn) mCherry fluorescent protein showed no clear difference to cytoplasmic and endocytic membrane structure within ventricle cardiomyocytes (S. Fig. 7). We were not able to determine whether nascent myofibrils were correctly anchored at the cell membrane after morpholino treatment. The resolution of analysis precluded an accurate assessment of (proto)-costamere structures.

Thus in the absence of *adprhl1* function, a sequence of myofibrillogenesis defects are observed, beginning with a loss of actin cytoskeleton rearrangement and delayed myosin production, that prevents early cell movements during chamber outgrowth. The defects subsequently cause wholesale failure to the architecture of the forming ventricle.

### 3.5 *Adprhl1* knockdown specificity confirmed by Western blot and RNA rescue experiment

Older stage 42 hearts were used to confirm the activity of the Adprhl1-e2i2MO morpholino by Western blot (antibody described in Materials and Methods). Two Adprhl1 protein bands were identified in control hearts; the 40 kDa. full length protein was the predominant species but a smaller, 23 kDa. protein was also evident. Importantly, both proteins were absent from hearts that received the *adprhl1* morpholinos ( S. Fig. 8A, C). Ordinarily, Adprhl1 protein was found within the heart at all stages of development. However, during later larval stages and in the juvenile and adult frog, the relative intensities of the bands were reversed and the 40 kDa. Adprhl1 protein became the minor form (S. Fig. 8B). The 40 kDa. Adprhl1 protein was restricted solely to heart tissue whereas some 23 kDa. protein was additionally detected within the tail (S. Fig. 8D). In spite of this, it is possible the 23 kDa. protein is also a genuine Adprhl1 species, since prolonged staining of embryos for *adprhl1* mRNA did yield a faint signal in somite and notochord tissues (S. Fig. 8E).

**Fig. 9:**
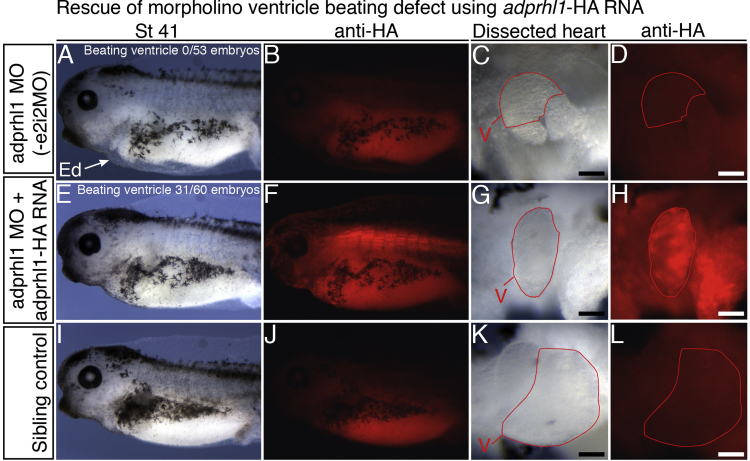
*Rescue of morpholino ventricle beating defect using adprhl1 RNA.* A–D: A tadpole injected with Adprhl1-e2i2MO into D-2/4 blastomeres, shown at stage 41. Left lateral (A, B) and ventral view of the dissected heart (C, D). E–H: A sibling tadpole co-injected with Adprhl1-e2i2MO and also adprhl1-HA RNA into D-2/4 blastomeres. The injection masses were 25 ng morpholino and 240 pg RNA. Immunological detection of HA-epitope gave a typical dorsal blastomere injection pattern (F) and signal in the ventricle of the dissected heart (H), which were absent in the tadpole injected solely with the morpholino (B, D). I–L: Comparable images of a sibling control tadpole. Red outlines show the position of the ventricle. The heart of the morpholino-injected tadpole was inactive, causing an oedema to form (A). The ventricle of the rescued tadpole was smaller than controls but actively contracted and no oedema developed (E). In this experiment, none of 53 tadpoles had a beating heart after injection of the morpholino while 31 out of 60 regained ventricular function with co-injection of adprhl1-HA RNA. Scale bars=100 µm. Ed, oedema; V, ventricle.

Because the Adprhl1-e2i2MO produced such a consistent heart defect, it was possible to confirm its specificity in rescue experiments where the morpholino was co-injected with a C-terminal HA-tagged *adprhl1* RNA (S. Fig. 9). The observed rescue was far from complete and hearts remained smaller than non-injected controls (S. Fig. 9C, G, K). Nonetheless, ventricular beating was frequently restored and consequently oedemas did not develop in stage 41 tadpoles (in this experiment, 31/60 rescued tadpoles had functional ventricles, compared with 0/53 in tadpoles that received the morpholino only).

### 3.6 Modelling Adprhl1 protein structure highlights key differences with the ADP-ribosylhydrolase active site

**Fig. 10:**
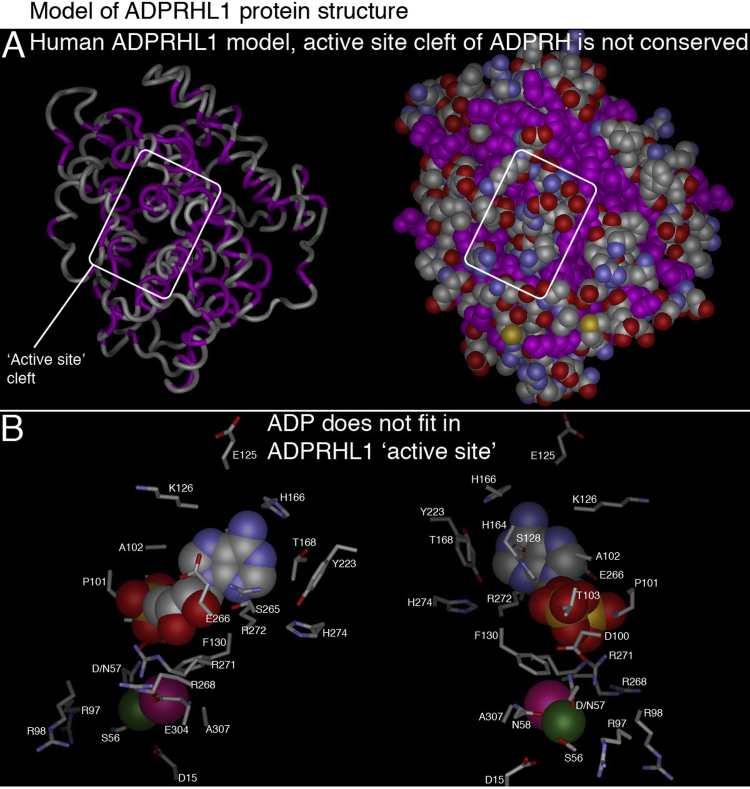
*Model of ADPRHL1 protein structure.* A: The sequence of human ADPRHL1, modelled onto the known structure of human ADPRH (PDB ID: 3HFW). The left panel shows the protein backbone while the right also shows the surface of amino acid side chains represented as space-filled spheres. Residues that are common to the two proteins are false-coloured magenta. The white rectangle marks the location of the active site cleft of ADPRH. The *Xenopus* Adprhl1 sequence produced essentially the same model. B: Detail of side chains from the active site cleft region that are conserved in ADPRHL1 proteins from all vertebrate species (coordinates used are from a model of the *Xenopus* sequence). Into this cleft, ADP has been forcibly inserted using the coordinates as found in ADPRH. However many side chains occlude favourable ADP binding (eg. D^100^, S^128^, F^130^, E^304^). In the left panel, the orientation of the cleft matches the view of the whole protein (A), while for the right panel, the cleft is rotated 90° around a vertical axis. The coordinates of a Mg^2+^ ion from structure -3HFW (green sphere) and a second (Mn^2+^) ion from related DRAG structure -2WOE (magenta sphere) are also depicted.

Our morpholino studies established that *adprhl1* was essential for proper myofibril assembly in forming heart chambers but did not indicate how it acted in this process. We speculated whether protein structural information could reveal insights into Adprhl1 function. Within the ADP-ribosylhydrolase family (pfam03747), crystal structures have been solved for human ADPRH (Kernstock et al., 2009), ADPRHL2 (Mueller-Dieckmann et al., 2006) and also DRAG, a key regulatory enzyme of nitrogen-fixing bacteria (Berthold et al., 2009, Li et al., 2009). We took advantage of sequence conservation among the homologs to model the Adprhl1 protein onto the known all-α-helical structure of human ADPRH (PDB ID: 3HFW). Both human and *Xenopus* Adprhl1 sequences were threaded onto the ADPRH structure. The entire sequence could be modelled, with no unstable domains or areas of steric conflict, the human and *Xenopus* species proteins folding equally well. Unsurprisingly, the resulting model illustrates how regions of extensive sequence identity between ADPRHL1 and ADPRH are mainly located in helices within the core of the proteins that are necessary to maintain structural integrity (S. Fig. 10).

The active site cleft in ADPRH that binds ADP-ribose has been identified and the crystal structure was even solved in complex with ADP. Additionally, the reaction mechanism of active ADP-ribosylhydrolases relies on a pair of divalent cations, magnesium for ADPRH and ADPRHL2, that are coordinated by aspartate residues located in N- and C-terminal portions of the enzyme (Berthold et al., 2009, Mueller-Dieckmann et al., 2006). Significantly, many amino acids present in the cleft are changed within ADPRHL1. Three of the four critical aspartates of ADPRH have been lost in mammalian ADPRHL1 (hADPRH D^56^, D^302^, D^304^ lost, only D^55^ is conserved), suggesting that ADPRHL1 cation binding may be compromised. Other key residues lost include a tyrosine and serines that stabilize the adenine and ribose groups (respectively) of ADP (hADPRH Y^263^, S^264^, S^269^, S^270^). In their place are residues that hinder substrate binding in the cleft (hADPRHL1 D^100^, S^128^, F^130^, E^304^) and thus it was not possible to dock ADP at the same position in the model of ADPRHL1 (S. Fig. 10B). Of particular note is the aspartate, D^100^, that could provide an alternative coordination site for metals but whose presence interferes with the phosphate groups of forcibly docked ADP. In the DRAG protein, the position of ADP-ribose binding is subtly altered (PDB ID: 2WOE) (Berthold et al., 2009), but again, using these coordinates could not accommodate ADP within the ADPRHL1 model (data not shown). Overall, the model and its altered active site cleft supports a current designation of ADPRHL1 as a pseudoenzyme. It is most unlikely to possess a direct ADP-ribosylhydrolase catalytic activity and so further experiments were designed to explore Adprhl1 function in the *Xenopus* heart.

### 3.7 Tight regulation of *Xenopus* Adprhl1 synthesis revealed by transgenic over-expression in the heart

**Fig. 11:**
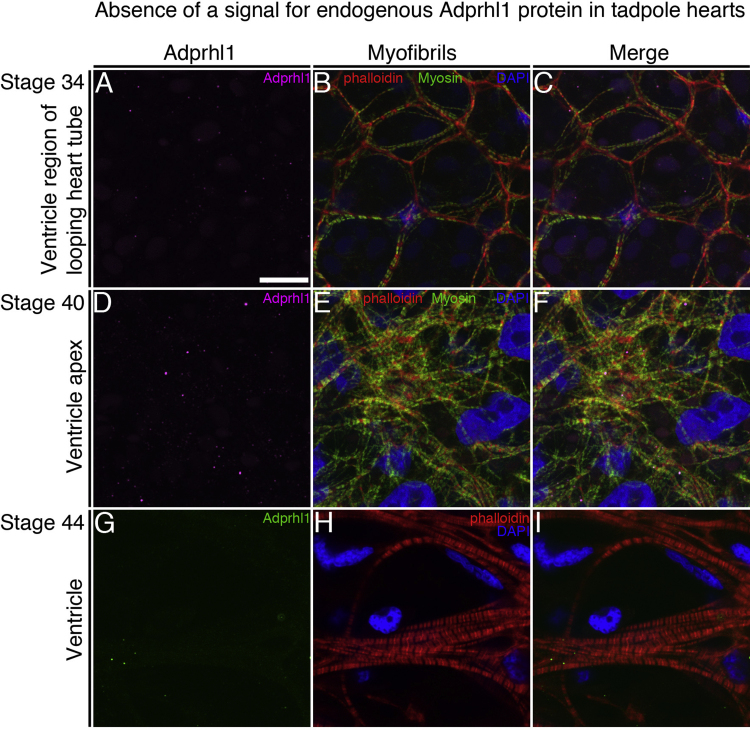
*Absence of a signal for endogenous Adprhl1 protein in tadpole hearts.* Despite the mRNA for *adprhl1* being an abundant molecule in embryonic hearts, endogenous levels of Adprhl1 protein are low and are not readily detected by immunocytochemistry. Scarce fluorescent dots observed using the Adprhl1 antibody can be detected in superficial optical sections of all embryonic tissues and are not specific. A–C: Cardiomyocytes in the ventricular region of a looping stage 34 heart tube. A: Anti-Adprhl1, using a far-red channel, fluorescent secondary antibody. B: Phalloidin, anti-myosin heavy chain and DAPI. C: Merge of four channels. D–F: Similar sequence of images of cardiomyocytes from the apex of the ventricle at stage 40. G–I: Stage 44 ventricular cardiomyocytes, this time using a different green channel, fluorescent secondary antibody to attempt Adprhl1 detection. Scale bar (A)=10 µm and applies to all images.

Despite the mRNA for *adprhl1* being an abundant molecule in embryonic hearts, Western blot analysis showed the protein to be relatively scarce (S. Fig. 8). Similarly, immunocytochemistry of hearts using the same antibody was not sufficiently sensitive to detect endogenous Adprhl1 protein *in situ* (S. Fig. 11). Furthermore, injection of *adprhl1* RNA into early embryos produced surprisingly little recombinant protein within the heart, irrespective of RNA amount, indicating possible post-transcriptional regulation of Adprhl1 expression.

**Fig. 13:**
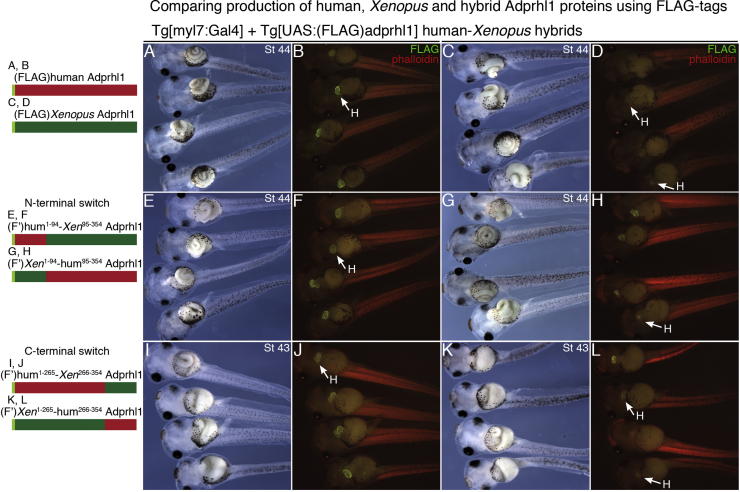
*Comparing production of human, Xenopus and hybrid Adprhl1 proteins in transgenic tadpoles with FLAG-tags.* The illustrations on the left indicate the composition of the human-*Xenopus* hybrid Adprhl1 proteins used in the experiment. A, B: Four representative transgenic tadpoles that express human ADPRHL1 protein in their hearts. Each tadpole carries the driver *Tg[myl7:Gal4]* transgene plus a new integration of a *Tg[UAS:(FLAG)human ADPRHL1]* responder transgene. Ventral view of stage 44 tadpoles (A) and matching fluorescence image (B) shows anti-FLAG immunocytochemistry (green) and phalloidin stain in the tail (red). C, D: Sibling tadpoles express little recombinant *Xenopus* Adprhl1 protein, despite having the same driver but a new integration of a *Tg[UAS:(FLAG)Xenopus adprhl1]* responder transgene. A weak signal for the FLAG epitope is only detected in a few myocardial cells with this *Xenopus*-species transgene. E–L: Transgenic tadpoles that express human-*Xenopus* hybrid Adprhl1 identify the 5′-cDNA (N-terminal) region as the site of regulation of the *Xenopus* protein. E, F: Tadpoles that carry the driver and a N-terminal switched, *Tg[UAS:(FLAG)human*^*1-94*^*-Xenopus*^*95-354*^*adprhl1]* responder transgene now produce detectable recombinant Adprhl1, because of the presence of the human sequence. G, H: Tadpoles with the driver and the converse *Tg[UAS:(FLAG)Xenopus*^*1-94*^*-human*^*95-354*^*adprhl1]* responder transgene still produce recombinant Adprhl1, although less (and in fewer animals) than the entirely human-species transgene (S. Table I). I, J: Tadpoles with the driver and a C-terminal switched, *Tg[UAS:(FLAG)human*^*1-265*^*-Xenopus*^*266-354*^*adprhl1]* produce expected levels of recombinant Adprhl1. K, L: Tadpoles with the driver and the converse *Tg[UAS:(FLAG)Xenopus*^*1–265*^*-human*^*266-354*^*adprhl1]* responder transgene express little recombinant Adprhl1, similar to the entirely *Xenopus*-species FLAG-tagged transgene. H, heart.

To investigate this possibility, we developed a binary system for transgene expression (S. Fig. 12), comprising a *Tg[myl7:Gal4]* myocardial-specific driver line and new *Tg[UAS:adprhl1]* responder transgenes, utilizing both the *Xenopus* and orthologous human *ADPRHL1* cDNAs. Significantly, tadpoles that contain both the driver and *Xenopus*-species responder transgenes do not produce detectable Adprhl1 protein in the heart, whereas those with the human *ADPRHL1* transgene always synthesize excess protein identified using the Adprhl1 antibody (Fig. 4A–F). A similar effect was also observed when N-terminal FLAG-tagged constructs were employed to ensure ready detection of recombinant proteins (S. Fig. 13A–D). The results suggest that during normal development, *Xenopus* Adprhl1 protein abundance is restricted within the forming heart and that the *Xenopus* transgene, consisting solely of coding cDNA sequence, is also subject to this negative regulation.

**Fig. 14:**
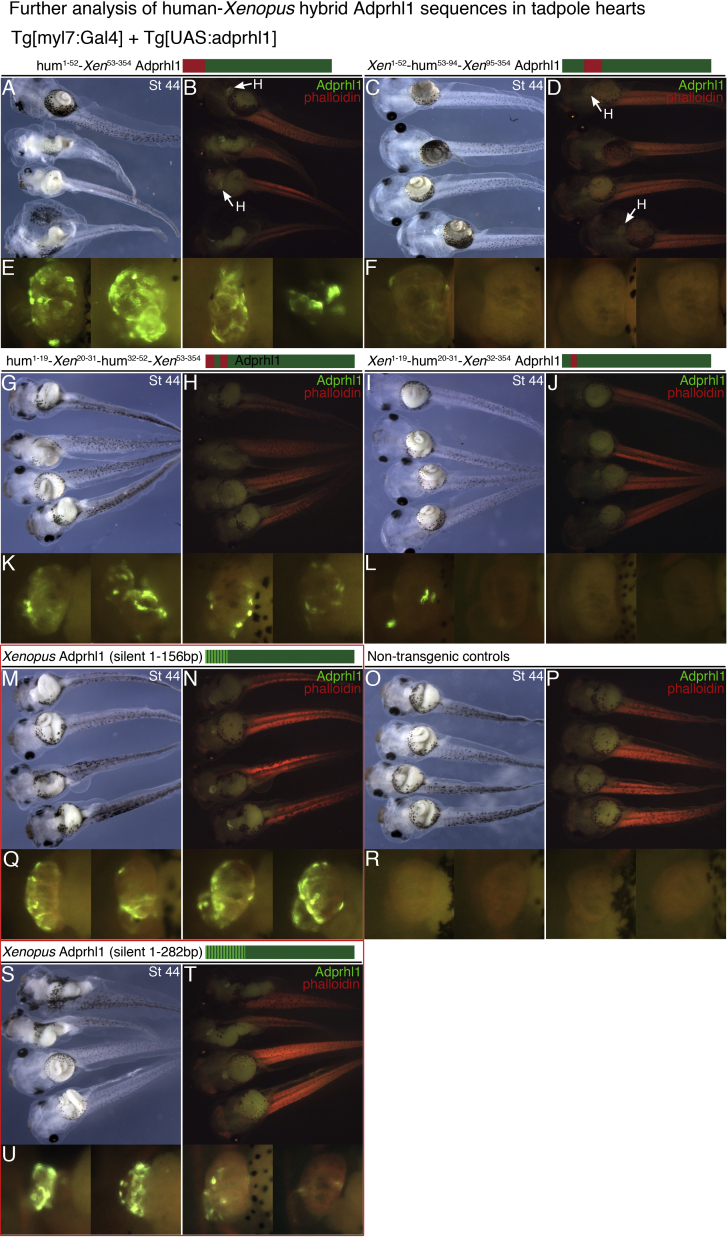
*Further analysis of human-Xenopus hybrid Adprhl1 sequences in tadpole hearts.* The illustrations at the top indicate the composition of six Adprhl1 hybrids tested for over-expression in transgenic tadpole hearts. Four are predominantly *Xenopus adprhl1* cDNAs but with small sequences switched to encode human-derived amino acids within the N-terminal region of the recombinant protein. Importantly, two constructs (M, N, Q, S, T, U) merely contain silent nucleotide changes within the 5′-portion of the *Xenopus* cDNA, such that the translated product remains identical to the endogenous Adprhl1 protein. A, B, E: Four tadpoles that carry the driver *Tg[myl7:Gal4]* transgene plus a new integration of a N-terminal switched, *Tg[UAS:human*^*1-52*^*-Xenopus*^*53-354*^*adprhl1]* responder transgene. Ventral view of stage 44 tadpoles (A) and matching fluorescence image (B) shows anti-Adprhl1 (green) and phalloidin stain in the tail (red). Detail view of the hearts (E) shows abundant hybrid Adprhl1. C, D, F: Tadpoles with the driver plus a Tg[UAS*:Xenopus*^1-52^-human^53-94^-*Xenopus*^95-354^ adprhl1] responder do not produce recombinant Adprhl1. G, H, K: The driver plus a complex Tg[UAS:human^1-19^-*Xenopus*^20-31^-human^32-52^-*Xenopus*^53-354^ adprhl1] responder transgene does yield detectable recombinant Adprhl1. I, J, L: The driver plus Tg[UAS*:Xenopus*^1-19^-human^20-31^-*Xenopus*^32-354^ adprhl1] does not produce recombinant Adprhl1 (just three positive cells within one heart). M, N, Q: The driver plus the *Tg[UAS:Xenopus adprhl1(silent 1-156bp)]* responder containing 36 silent nucleotide changes does produce recombinant Adprhl1 (also shown in Fig. 4). O, P, R: Non-transgenic control tadpoles that are siblings to those in (M, N, Q). S, T, U: Additionally, the driver plus the *Tg[UAS:Xenopus Adprhl1(silent1-282bp)]* responder with 69 silent nucleotide changes also produces recombinant Adprhl1. The Adprhl1-peptide antibody binds at aa residues 249–266. H, heart.

The sequences responsible for *Xenopus* Adprhl1 regulation and human ADPRHL1 production were mapped by testing constructs that switched nucleotide sequences between the two species in order to encode human-*Xenopus* hybrid proteins (S. Figs. 13 and 14). A hybrid comprising the first 94 amino acids of human ADPRHL1 (1-282 bp) combined with residues 95-354 of *Xenopus* Adprhl1 escaped the regulation and did accumulate in transgenic hearts (S. Fig. 13E, F). As the size of the exchanged N-terminal sequence was further reduced (1-156 bp, 1-52 aa), so the frequency of transgenic tadpoles producing detectable Adprhl1 became lower (S. Fig. 14B, E, S. Table I). Ultimately, transgenes that contain silent nucleotide changes and yet still produce a translated protein identical to *Xenopus* Adprhl1 also yielded a significant signal in transgenic hearts (36 changes within 1-156 bp; Fig. 4G, H, K) (69 changes in 1-282 bp; S. Fig. 14S–U). Thus the 5′-cDNA sequence, rather than the amino acid sequence of the protein, was decisive for negative regulation of transgene expression.

**Fig. 15:**
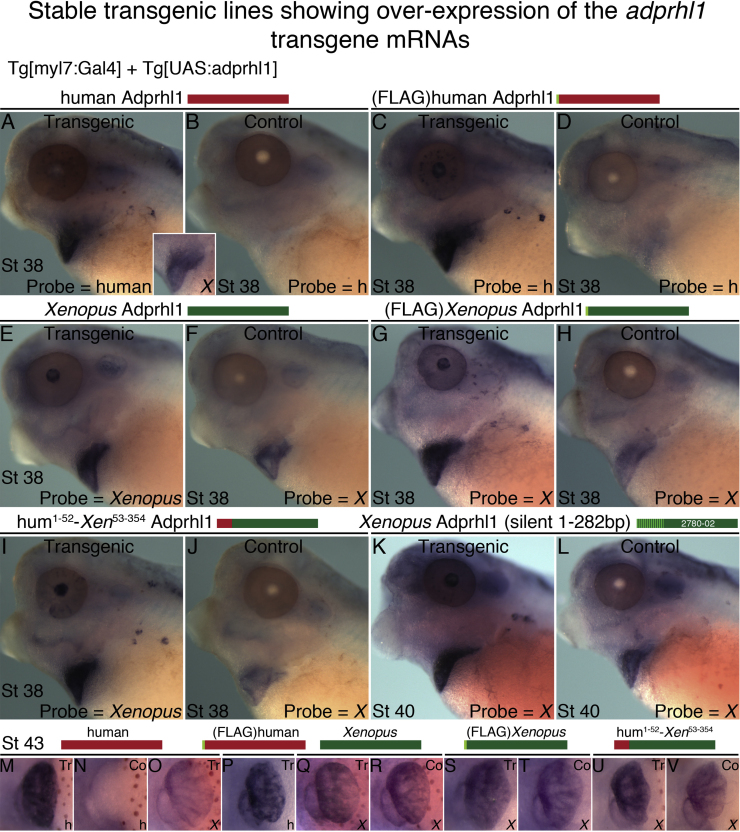
*Stable transgenic lines showing over-expression of the adprhl1 transgene mRNAs.* A–L: Cardiac *adprhl1* mRNA expression in embryos representing six stable *Tg[UAS:adprhl1]* transgenic lines. A, B: Stage 38 tadpole containing *Tg[myl7:Gal4]* driver and *Tg[UAS:human ADPRHL1]* responder transgenes (A), plus a sibling control (B), after *in situ* hybridization for the human *ADPRHL1* RNA. The transgenic tadpole produces *ADPRHL1* mRNA in the heart whereas the control does not. The transgenic heart in the inset shows a weaker signal for the endogenous *adprhl1* mRNA using a *Xenopus* probe of equivalent size (Materials and Methods). C, D: Comparable human mRNA synthesis from the *Tg[UAS:(FLAG)human ADPRHL1]* transgene. E, F: *Xenopus adprhl1* mRNA over-expression from the *Tg[UAS:Xenopus adprhl1]* transgene. G, H: The *Tg[UAS:(FLAG)Xenopus adprhl1]* transgene, which yields stronger expression than the untagged construct. I, J: Expression driven by the hybrid *Tg[UAS:hum*^*1-52*^*-Xen*^*53-354*^*adprhl1]* transgene. K, L: Expression from the *Tg[Xenopus adprhl1(silent1-282bp)]* transgene that contains silent nucleotide changes, shown in a stage 40 tadpole. M–V: By stage 43, the signal for endogenous *adprhl1* mRNAs appears stronger and only transgenes with human *ADPRHL1* nucleotide sequence sustain elevated mRNA levels. Five of the stable lines are represented, with ventral views of heart ventricles shown. A–L: All transgenic embryos additionally express transient *adprhl1* mRNA within the eye lens, because the *Tg[myl7:Gal4]* driver line has no insulating sequences to separate the γ−crystallin promoter (S. Fig. 12). Eye *in situ* hybridization signals for the responder mRNA can therefore be used to identify transgenic embryos. The lower-right of each panel lists the species of hybridization probe used. Tr, binary transgenic; Co, control.

**Fig. 16:**
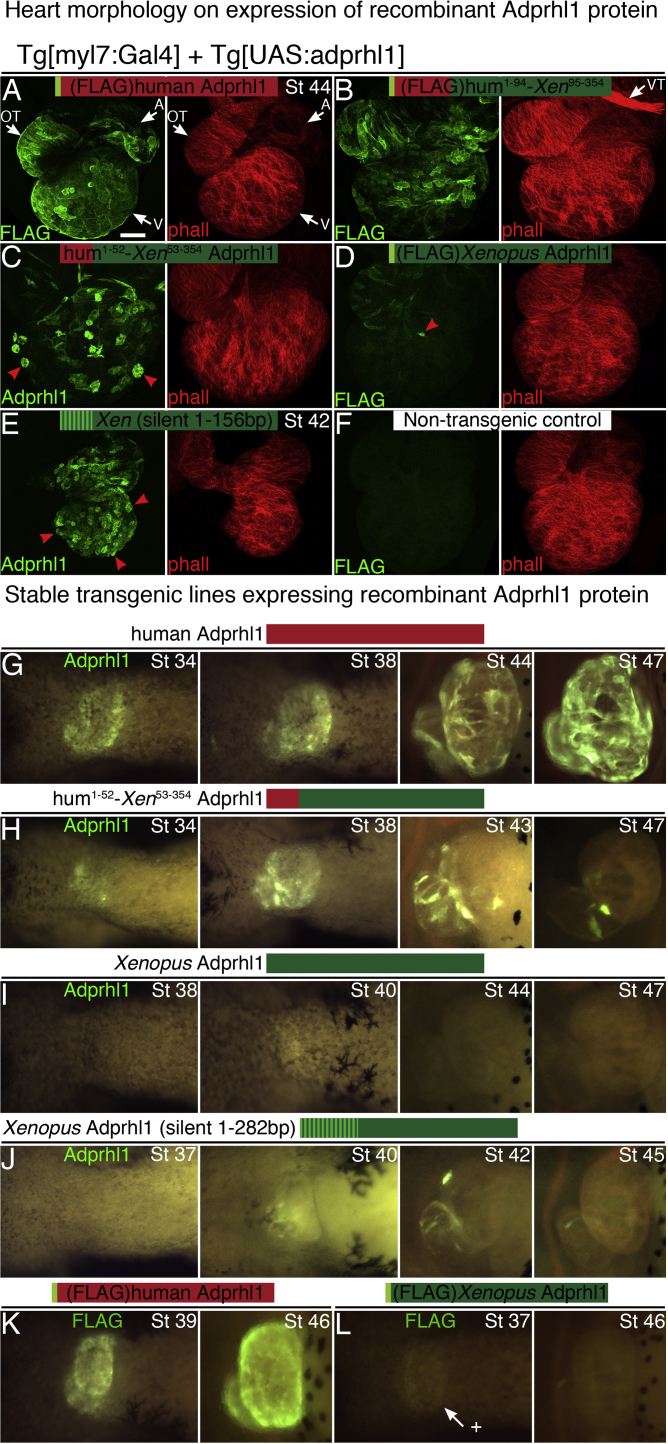
*Expression of recombinant Adprhl1 protein in transgenic hearts.* A–F: Expression of Adprhl1 protein in the heart of founder generation transgenic tadpoles. Each carries the *Tg[myl7:Gal4]* driver plus a new integration of an *adprhl1* responder. A: *Tg[UAS:(FLAG)human ADPRHL1]*. B: *Tg[UAS:(FLAG)hum*^*1-94*^*-Xen*^*95-354*^*adprhl1]*. C: *Tg[UAS:hum*^*1-52*^*-Xen*^*53-354*^*adprhl1]*. D: *Tg[UAS:(FLAG)Xenopus adprhl1]*. E: *Tg[UAS:Xenopus adprhl1(silent 1-156bp)]*. F: Non-transgenic sibling control heart. Dissected hearts are shown, anterior view, at stage 44 (A–D, F) and stage 42 (E). Cells with a rounded morphology that protrude from the ventricle surface, found in hybrid and silent mutation transgenics are indicated (red arrowheads). The heart (E) represents a transgenic containing large numbers of *Xenopus* Adprhl1-positive cells and thus shows abnormal cardiac chamber development. A, B, D, F: Anti-FLAG (green) and phalloidin actin stain (red). C, E: Anti-Adprhl1 and phalloidin. Scale bar (A)=100 µm. V, ventricle; A, atrium; OT, outflow tract; VT ventral transverse skeletal muscle, that was not dissected away from the heart (B). G–L: Adprhl1 protein expression in embryos representing six stable *Tg[UAS:adprhl1]* transgenic lines. G: *Tg[UAS:human ADPRHL1]*. H: *Tg[UAS:hum*^*1-52*^*-Xen*^*53-354*^*adprhl1]*. I: *Tg[UAS:Xenopus adprhl1]*. J: *Tg[UAS:Xenopus adprhl1(silent 1-282bp)]*. K: *Tg[UAS:(FLAG)human ADPRHL1]*. L: *Tg[UAS:(FLAG)Xenopus adprhl1]*. The developmental stage series highlights the transient nature of Adprhl1 over-expression with the hybrid and silent mutation transgenes. Ventral views, anterior to the left.

Stable lines were established for key *adprhl1* transgenes (S. Fig. 12D), which confirmed the original observations. Moreover, they enabled study of transgene mRNA expression within F1-generation embryos. Importantly, irrespective of 5′-sequence, all transgenes caused up-regulation of *adprhl1* mRNA throughout the heart, detected around stage 38 (S. Fig. 15A–L). Nonetheless, despite the initial induction, by stage 43, only the human *ADPRHL1* transgene maintained elevated mRNA expression. At stage 43, tadpoles with the *Xenopus*-sequence transgene showed expression identical to control hearts, while the hybrid (1-52 aa) and the silent mutation transgenes produced only subtle mRNA increases (S. Fig. 15M–V). Adprhl1 protein synthesis in the stable lines was essentially the same as in founder generation animals. Excess production was sustained beyond stage 47 for human ADPRHL1, but was only transient for the stable lines of hybrid and silent mutation transgenes (S. Fig. 16G–L).

Transgene experiments therefore suggest control of *Xenopus* Adprhl1 synthesis acts at two levels during mRNA biogenesis. During early chamber outgrowth stages, translational inhibition can restrict Adprhl1 production, despite the presence of an abundant mRNA pool. Later at stage 43, the abundance of these transgene mRNAs is also affected. During normal *Xenopus* heart development, it is noteworthy that stage 43 sees a reduction in abundance of 40 kDa full length Adprhl1 relative to the smaller 23 kDa. protein species. Since the transgenes encode 40 kDa. Adprhl1, it is possible they were also susceptible to a mechanism that induces a switch in endogenous Adprhl1 forms.

### 3.8 Adprhl1 produces disarrayed myofibril patterns with branches at actin-Z-disc boundaries

Having established regulatory constraints for transgenic production *in vivo*, we were able to examine the consequence of Adprhl1 (40 kDa) over-expression within the embryonic heart. Transgenic tadpole hearts were subjected to a detailed study of myofibril patterns, concentrating on the ventricular chamber but also analyzing atrial, atrio-ventricular canal and outflow tract regions. Stage 44 was chosen for analysis of founder generation transgenics, while the stable lines permitted study of a range of developmental stages. Comparisons were made between hearts that produced different Adprhl1 variants: (1) human ADPRHL1, (2) the N-terminal human-*Xenopus* Adprhl1 hybrids (hybrid-1-94 and hybrid-1-52 aa), (3) those hearts identified containing positive cells for FLAG-tagged *Xenopus* Adprhl1 and most tellingly, (4) natural *Xenopus* Adprhl1 protein produced from the transgenes with silent mutations (S. Table I, S. Fig. 12). A range of myocardial abnormalities was observed with, unsurprisingly, the *Xenopus*-species protein giving the more extreme effect on tadpole heart myofibrils.

Hearts containing human ADPRHL1 developed normally, with correctly proportioned chambers, outflow tract and identical myofibril patterns to controls, suggesting the human protein had no interfering activity when synthesized in tadpole hearts. Conversely, the hybrid-1-94 aa Adprhl1 protein was associated with specific cardiomyocyte structural changes. Within the ventricular wall, myofibrils growing with excess hybrid-1-94 aa Adprhl1 produced extensively branched, striated actin filaments (Fig. 5E, G). The point at which sarcomeres divided was always a modified Z-disc, as identified by the bright stripe of phalloidin-actin staining (Fig. 5G). Branched myofibrils also occurred with the hybrid-1-52 aa, the FLAG-tagged *Xenopus* and natural *Xenopus* Adprhl1 proteins. Moreover, these proteins additionally yielded some cells with a severe myofilament defect, whose prevalence was highest with the native *Xenopus* Adprhl1. In these cells, phalloidin stained circular branch points with numerous thin (non-striated) actin filaments radiating outwards from the foci (Fig. 6C, G). The extreme myofilament patterns gave cells a rounded appearance and they protruded from the ventricle surface, both outwards and inwards towards the lumen (S. Fig. 16A–F). Such spherical cells, excluded from the functional ventricular wall, probably represented the culmination of the aberrant branching process observed in all the hearts.

In the stable lines expressing hybrid and the silent mutation transgenes, functional embryonic hearts were ultimately formed. This occurred because these transgenes did not maintain elevated *adprhl1* mRNA expression by stage 43 and so recombinant Adprhl1 protein subsequently returned to endogenous levels (S. Fig. 16G–L). Consequently, during analysis, there was variability in Adprhl1 production by individual cells, often between adjacent cardiomyocytes, which did allow comparison of positive and negative regions within the same heart (Fig. 5D, F). Some founder generation tadpoles were identified that contained large numbers of *Xenopus* Adprhl1-positive, spherical cardiomyocytes and these always resulted in abnormal development of the embryonic heart chambers (S. Fig. 16E).

Increased myofibril branching was recorded in all regions of the heart, since the *myl7* promoter of the driver transgene is active in all myocardial cells and not merely biased towards the chambers. Sarcomere divisions caused by Adprhl1 were detected in non-chamber myocardium of the A-V canal (Fig. 6I–L) and within atrial cells (Fig. 6M–P). Moreover, staining for sarcomeric α-actinin confirmed it localized to the branched Z-disc structures (Fig. 6L, P).

### 3.9 Adprhl1 can localize to cardiac sarcomeres, adjacent to the Z-disc and to the H-zone

Recombinant Adprhl1 usually showed a widespread distribution within the cardiomyocyte. However, using detection of the N-terminal FLAG-tagged Adprhl1, it was possible to find cells where Adprhl1 actually associated with the sarcomeres. In cells where there was little actin branching (or perhaps had not yet altered their filament structure), FLAG-tagged human ADPRHL1 and also *Xenopus* Adprhl1 were observed in a striated pattern (Fig. 7). Adprhl1 localized to two clear stripes on either side of the Z-disc (Fig. 7D, green arrowhead) and also to a diffuse stripe marking the H-zone of the sarcomere (Fig. 7D, E, I, white arrowheads). These localizations, potentially representing actin boundaries, provide evidence that 40 kDa. Adprhl1 may alter myofibril structure through a direct action on the sarcomere.

## 4 Discussion

We have identified *adprhl1* as an important gene that acts during cardiogenesis by performing gene knockdown and transgenic over-expression studies using embryos of the frog, *Xenopus laevis*. The expression of *adprhl1* mRNA in the embryonic *Xenopus* heart is biased towards actively growing, chamber myocardium. Moreover, cardiac expression of *Adprhl1* mRNA is also conserved in mouse embryos. It has previously been observed that *ADPRHL1* is among mRNAs that are induced as human embryonic stem cells are differentiated *in vitro* towards a cardiac fate (Beqqali et al., 2006) but, until now, the function of Adprhl1 in the forming vertebrate heart has never been addressed.

Our studies show that *adprhl1* is a gene whose activity is essential for assembly of contractile myofibrils and outgrowth of the cardiac chambers. Nonetheless, when synthesis of full length (40 kDa) Adprhl1 is forced in tadpoles by the introduction of transgenes, it does not simply result in production of additional myofibrils. Rather, over-expression also disrupts myofibrillogenesis. Engineered human-*Xenopus* hybrid proteins cause extensive branching of myofibrils in our assay, with sarcomere division occurring at the actin-Z-disc boundary. Excess native *Xenopus* Adprhl1 can produce even greater effects; the actin filament network appears merely as thin threads connected to numerous circular branch points, or has regressed to the extent that just these punctate dots remain visible.

The potent action of Adprhl1 on cardiomyocyte structure may at least in part account for our finding that detection of the protein in the developing heart is challenging and that a negative regulatory mechanism restricts its synthesis. We presume that in normal development, either a very low level of protein is sufficient for Adprhl1 function or that a transient signal releases this control and permits synthesis of *Xenopus* Adprhl1 as the heart chambers grow.

Why does ventricle formation fail in the absence of Adprhl1? Distinct patterns of cardiomyocyte growth define the shape of the emerging ventricle, plus cell proliferation also occurs within forming chambers. During the transition from a tubular heart, cardiomyocytes in the presumptive ventricle region elongate with unique trajectories such that they transiently appear aligned into a rosette pattern (Fig. 3J). This early growth, which is biased to the left side, breaks the left-right symmetry, contributes to the characteristic looped shape and defines the position of the apex. Of course, cardiomyocyte growth is tied to myofibril assembly. These chamber cells produce myofibrils whose predominant orientation will extend across the ventricle width (perpendicular to base-to-apex axes, Fig. 3K, M). Ultimately, the embryonic ventricle's size and form is governed by the extent of cells growing with chamber trajectories on the outer surface, combined with successive development of trabecular cardiomyocytes on the lumenal side. Trabeculae myofibrils orient along the ventricle length (parallel to base-to-apex axes, Fig. 3K, N).

Knockdown of *adprhl1* is associated with myofibrillogenesis defects from the earliest stages of ventricle outgrowth. Actin filaments remain fixed at the cell cortex, chamber cardiomyocytes do not elongate and myosin synthesis is delayed, processes that are all interlinked. It is difficult to establish whether Adprhl1 acts directly on myofibrils from the morpholino experiments alone, or if cell signalling upstream fails to initiate the programme. The localization of recombinant Adprhl1 protein in transgenic hearts does nonetheless suggest a direct action on myofibrils and actin boundaries in particular. Given the dynamics of actin fibres, loss of Adprhl1 could impair filament degradation or initiation of new actin polymerization and conceivably yield similar myofibril deficits.

Aside from the myofibrillogenesis and cell polarity defects, morphant heart tubes at stage 33 have a normal external morphology and contain equivalent numbers of cardiomyocytes. However, by stage 37 and beyond, hearts developing without *adprhl1* function become badly malformed. Myofibrils that do eventually form in the ventricle are short and disarrayed, while their cardiomyocytes appear disordered. We believe the lower cell numbers measured on the surface of small morphant ventricles reflects a loss of structural organization of the cardiomyocytes caused as a consequence of the earlier defects. The cumulative effect of heart development proceeding without a working heart beat might also prevent continued recruitment of cardiac progenitors into the heart chambers.

How might regulation of Adprhl1 synthesis be achieved? Our experiments with *adprhl1* transgenes point towards translational control occurring as the ventricle grows, while there is also evidence of repression, post-stage 43, acting on *adprhl1* mRNA abundance that coincides with an apparent shift towards a smaller Adprhl1 protein form. The mechanism targets the 5′-coding sequence of the *Xenopus adprhl1* mRNA, since the inhibition of transgene expression can be evaded by substituting 36 silent nucleotide changes in the first coding 156 bp. A number of the transgenes included a 5′-inserted FLAG tag sequence that we also suspect of subtly reducing effective targeting (S. Table I). This might suggest the immediate sequence surrounding the translation initiating ATG to be the most critical for inhibition to occur. However, comparing the targeted *Xenopus* sequence with the human *ADPRHL1* that is not regulated in the assay, there are just two nucleotide differences in the 24 bases immediately downstream of the ATG (S. Fig. 12E), indicating that a larger region of 5′-sequence is required.

In the mouse, conditional deletion of the miRNA-processing enzyme Dicer1 in myocardial tissues does cause disarrayed myofibril structure and post-natal cardiac lethality (Chen et al., 2008). However, involvement of miRNAs in control of Adprhl1 seems unlikely, since they invariably elicit their effects by binding untranslated regions of mRNAs. Moreover, their action tends to modulate or fine-tune gene expression levels, rather than imposing an absolute block on synthesis (reviewed Espinoza-Lewis and Wang (2012)).

Post-transcriptional regulation of gene expression can occur at many levels, from alternative RNA splicing, mRNA transport, localization and stability, as well as translational inhibition. In the heart, RNA binding proteins (RBP) that mediate these processes have been shown to affect cardiogenesis (Blech-Hermoni and Ladd, 2013). At present, much less is known regarding which cardiac gene mRNAs are targeted and at what point during their processing. RBPs with high expression in the heart include Hermes, whose over-expression in *Xenopus* embryos causes early defects to cardiogenesis (Gerber et al., 2002), plus Fxr1 (Huot et al., 2005), whose action leads to translational inhibition of talin2 and desmoplakin in mouse cardiac muscle (Whitman et al., 2011). A further example is the Champ RNA helicase, whose expression in mouse embryos is biased towards the chamber trabeculae, but again, target RNAs have not been identified (Liu et al., 2001). Thus, there are many RBP candidates that can be explored as potential mediators of Adprhl1 regulation in the forming heart.

Using the cardiac-Gal4/UAS system, expression of responder transgenes commences upon myocardial differentiation (stage 28), with fluorescence from a GFP reporter becoming visible in the linear heart tube (stage 32) (data not shown). This suggests that in our over-expression experiments, the entire process of chamber outgrowth and its associated myofibrillogenesis occur in the presence of increasing concentration of 40 kDa. Adprhl1. The resulting tadpole hearts were analyzed at stage 44, once the ventricle has formed. At this stage, early trabecular ridges have formed and the initially opaque *Xenopus* tissues have become transparent, facilitating improved microscopy. The appearance of unusual myofibril morphology thus reflects the prolonged action of Adprhl1 during approximately three days of cardiac development.

How might over-expression of Adprhl1 cause the punctate actin patterns and myofibrillar branching? One possibility is that the structures are merely a consequence of disrupted myofibrillogenesis. They could represent a stalled, intermediate step during rearrangement of the actin cytoskeleton. Alternatively, they could result from excessive initiation (nucleation) of actin thin filament polymerization. In current models of myofibrillogenesis, the starting points for assembly are aggregations of α-actinin and actin (Z-bodies) that are associated with the cell membrane at integrin adhesion sites (proto-costameres) (Sparrow and Schock, 2009). Current understanding of nucleation and initial assembly of actin thin filaments remains incomplete. As an example, the muscle formin, Fhod3, which has been implicated as a key nucleator of actin, actually reduces actin elongation when tested *in vitro* (Iskratsch et al., 2010, Taniguchi et al., 2009). Nevertheless, we may speculate that myofibril abnormalities occur because excess Adprhl1 continually supports attachment of nascent filaments to Z-bodies, or causes defective processing so that multiple actin filaments are allowed to elongate with differing orientations from a maturing Z-disc. This might conceivably reflect a normal role for Adprhl1 in enabling formation of the unique three-dimensional myofibril mesh that distinguishes cardiac from skeletal muscle.

A second possibility is that branched myofibrils actually arise during normal development of the cardiac chamber and that their frequency has been increased by Adprhl1 over-expression. Evidence from embryonic zebrafish hearts now supports the view that branching naturally occurs within discrete chamber regions. A branched network of myofibrils has been observed at the outermost surface of ventricle cardiomyocytes (Reischauer et al., 2014). At this location, they may function to couple laterally positioned costameres and form bridges to deeper lying contractile myofibrils. Branches appeared transiently in the atrium, before myofibrils were bundled together into larger, straight fibres. Moreover, branches were also reported towards the distal ends of myofibrils in zebrafish ventricle trabeculae (Reischauer et al., 2014). Within the *Xenopus* ventricle, trabeculae have many bifurcations as the ridges extend from the A-V canal into the chamber apex. Thus branching could enable myofibrils to transmit contractile force from one trabecular cardiomyocyte on to two diverging ridges.

The developing *Xenopus* heart offers many advantages for those studying the link between chamber formation and myofibrillogenesis, in terms of both its large size and relative complexity. Nonetheless, a definitive atlas of cardiac myofibril structure for *Xenopus* chambers remains to be produced. Consequently, while our over-expression study summarises the analysis of a very large number of transgenic hearts, the increased branching of myofibrils caused by Adprhl1 remains a qualitative observation.

One further complexity in both *Xenopus* and mouse hearts is the existence of a 23 kDa. protein identified by the Adprhl1 antibody, in addition to the full length 40 kDa. Adprhl1 we have studied. Both proteins are sensitive to *adprhl1* knockdown with splice-morpholinos. However, while 40 kDa. Adprhl1 is found solely in heart muscle, the 23 kDa. protein can also be detected within the tadpole tail. The smaller protein is unlikely to be a product of the other ADP-ribosylhydrolase family members, given the unique sequence selected for the antibody. While its identity has not yet been proven, it could derive from a distinct *adprhl1* primary mRNA transcript or be a processed Adprhl1 fragment.

The concept of a pseudoenzyme comes from study of the large family of protein kinases. Following duplication of a gene, evolution-driven variation has produced proteins that have lost essential residues of their catalytic active site. Hence pseudokinases have arisen that redeploy a substrate binding activity for a new purpose, or that associate with and modify the conformation of a relative kinase that retains enzymatic activity (Leslie, 2013). It is a useful comparison to make with Adprhl1. Could cardiac Adprhl1 serve to modulate the activity of Adprh or Adprhl2, or more likely, bind to proteins that are shared targets of the active enzymes? In some circumstances, ADP-ribosylation can alter filament protein dynamics. Bacterial toxin ADP-ribosyltransferases are known to target cytoskeletal proteins of infected cells and ADP-ribosylation prevents polymerization of non-muscle actin, vimentin and desmin filaments (Icenogle et al., 2012). ADP-ribosylated actin (at arg^177^) even acts as a capping protein on existing filaments and we find it intriguing why muscle α-actins proved less susceptible to modification by toxins in these studies (Hochmann et al., 2006). Thus an indirect role for Adprhl1 to bind proteins that are themselves subject to ADP-ribosylation circuits remains a distinct possibility. The different activities of Adprhl1 that contain human sequences in our tadpole over-expression assay may be due to altered rates of binding to such target proteins.

The precise function of Adprhl1 may emerge by studying its potential association with the Z-disc borders, since this will offer a list of potential protein targets. The presence of two stripes of Adprhl1 on both sides reflects the symmetry within the Z-disc (reviewed Luther (2009)), found in the anti-parallel dimers of α-actinin and also the barbed ends of opposing actin filaments. The additional stripe of Adprhl1 marking the H-zone could represent a distinct association or potentially indicate an Adprhl1 binding partner is found at both Z-disc and M-band regions. A first step towards identifying precise targets will be to map domains within the Adprhl1 sequence that mediate the sarcomeric localization.

## Figures and Tables

**Fig. 3:**
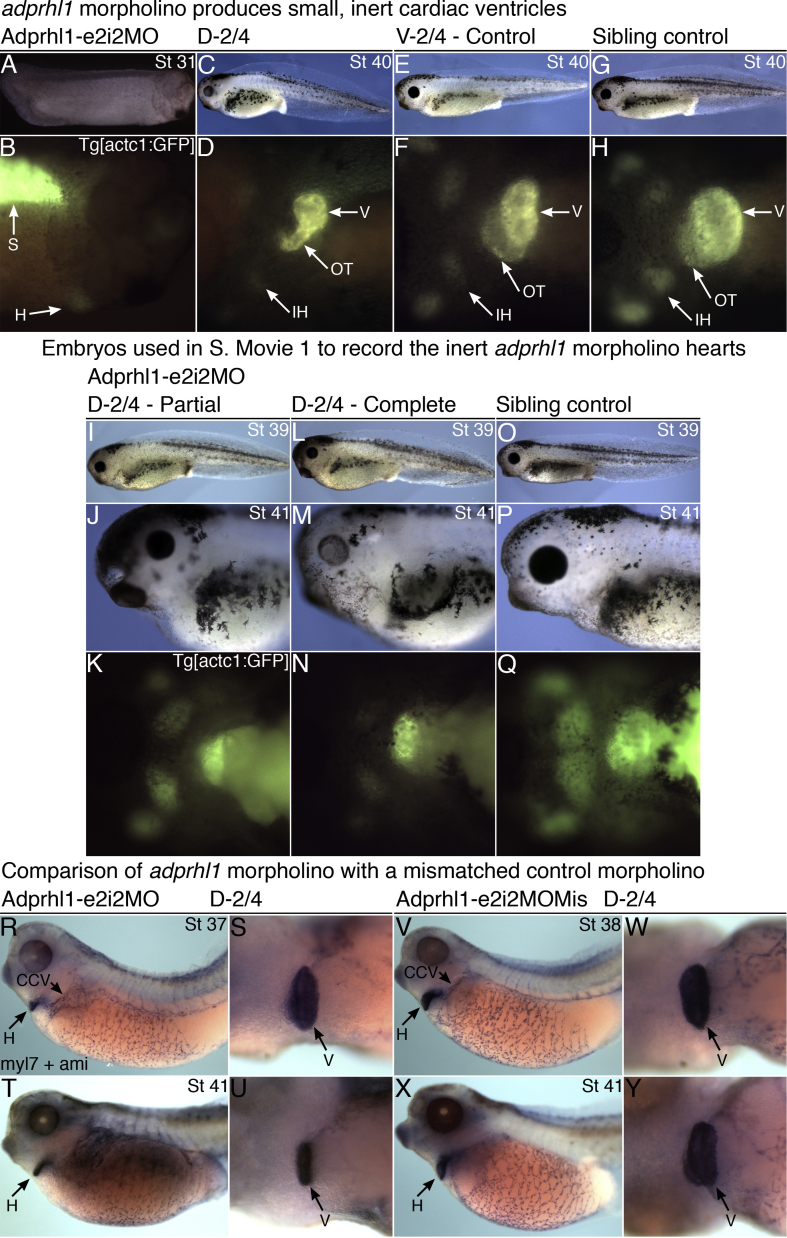
*Adprhl1 morpholino produces small, inert cardiac ventricles.* A–D: A single tadpole injected with Adprhl1-e2i2MO into D-2/4 blastomeres, shown at stage 31 (A, B) and stage 40 (C, D). The small heart of this tadpole did not contract. E, F: Control tadpole that received the same morpholino into V-2/4 blastomeres. G, H: Sibling control tadpole. Hearts were visualized using the *Tg[actc1:GFP]* (cardiac actin) reporter transgene. Ventral view of hearts, anterior to left (D, F, H), Right-lateral view (B). H, heart; S, somites; V, ventricle; OT, outflow; IH, interhyoid facial muscle. I–Q Embryos used in S. Movie 1 to record the inert *adprhl1* morpholino hearts. I-K: Tadpole with Adprhl1-e2i2MO into D-2/4 blastomeres that has a partial heart beat defect. L–N: A D-2/4-morpholino tadpole with completely inert heart. O-Q: Sibling control. Embryos appear normal at stage 39 (I, L, O) while at stage 41 (J, M, P) when the movies were recorded, oedemas are beginning to form in those with the morpholino. Ventral view of *Tg[actc1: GFP]* fluorescence (K, N, Q). R–Y: Comparison of *adprhl1* morpholino with a mismatched control morpholino. R–U: Tadpoles injected with Adprhl1-e2i2MO into D-2/4 blastomeres showing expression of cardiac *myl7* and vascular *ami* mRNAs. At stage 37 (R, S), there are no apparent differences in cardiac morphology but at stage 41 (T, U), the ventricle is clearly smaller than controls. The oedema does cause a higher background hybridization stain to develop (T). V-Y: Control sibling tadpoles injected with the Adprhl1-e2i2MOMis morpholino that contains 5 mismatched bases. Ventral views, anterior to left (S, U, W, Y). One indirect sign of cardiac dysfunction that can be detected prior to oedema is the broader pattern of *ami* expression in the common cardinal vein (CCV) region (compare R and V). This is also observed in Fig. 2(A, E, I).

**Fig. 4:**
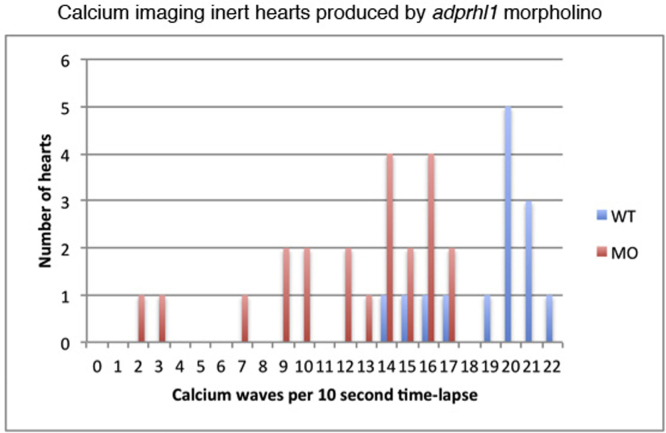
*Calcium imaging inert hearts produced by adprhl1 morpholino.* Chart showing the number of calcium waves recorded during 10 s movie clips in 22 different Adprhl1-e2i2MO injected, inert tadpole hearts compared with 14 wildtype controls at stage 42. The frequency of Ca^2+^ transients is reduced with the morpholino, but is not the primary cause of the cardiac functional defect. A lower 20 ng mass of morpholino was used in this experiment to accommodate co-injection of the RNA into embryos. Thus a reduced proportion of tadpoles developed the complete phenotype associated with the Adprhl1-e2i2MO (20 out of 42 tadpoles injected, 48%).

**Fig. 5:**
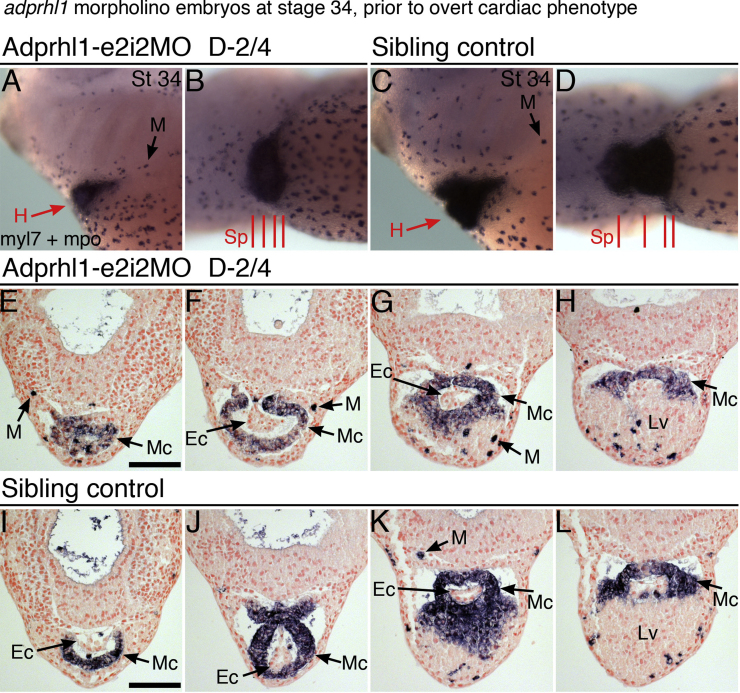
*Adprhl1morpholino embryos at stage 34, prior to overt cardiac phenotype.* A, B, E–H: The heart of a stage 34 embryo injected with Adprhl1-e2i2MO into D-2/4 blastomeres, before the cardiac phenotype becomes apparent. Expression of *myl7* in the myocardium and *mpo* in migrating embryonic macrophages are shown in left lateral (A) and ventral (B) views. The red lines (B) mark the position of four transverse sections cut through the heart outflow (E), ventricle (F), atrial (G) and inflow regions (H). C, D, I–L: Comparable images of a sibling embryo. In this experiment, half the cohort of injected embryos were allowed to develop on to stage 40 to confirm the morpholino’s subsequent action on heart formation and function. The macrophage marker was included to prove that impaired endocardial development noted at later stages did not originate from defective macrophage migration from the heart-forming region. Stage 33–34 marks the onset of cardiac contractions in control embryos. The only difference noted in morpholino injected embryos was within the outflow region, which did not extend rostrally but rather occupied a dorsal position. Scale bars=100 µm. H, heart; M, macrophage; Sp, section position; Mc, myocardium; Ec, endocardium; Lv, liver.

**Fig. 6:**
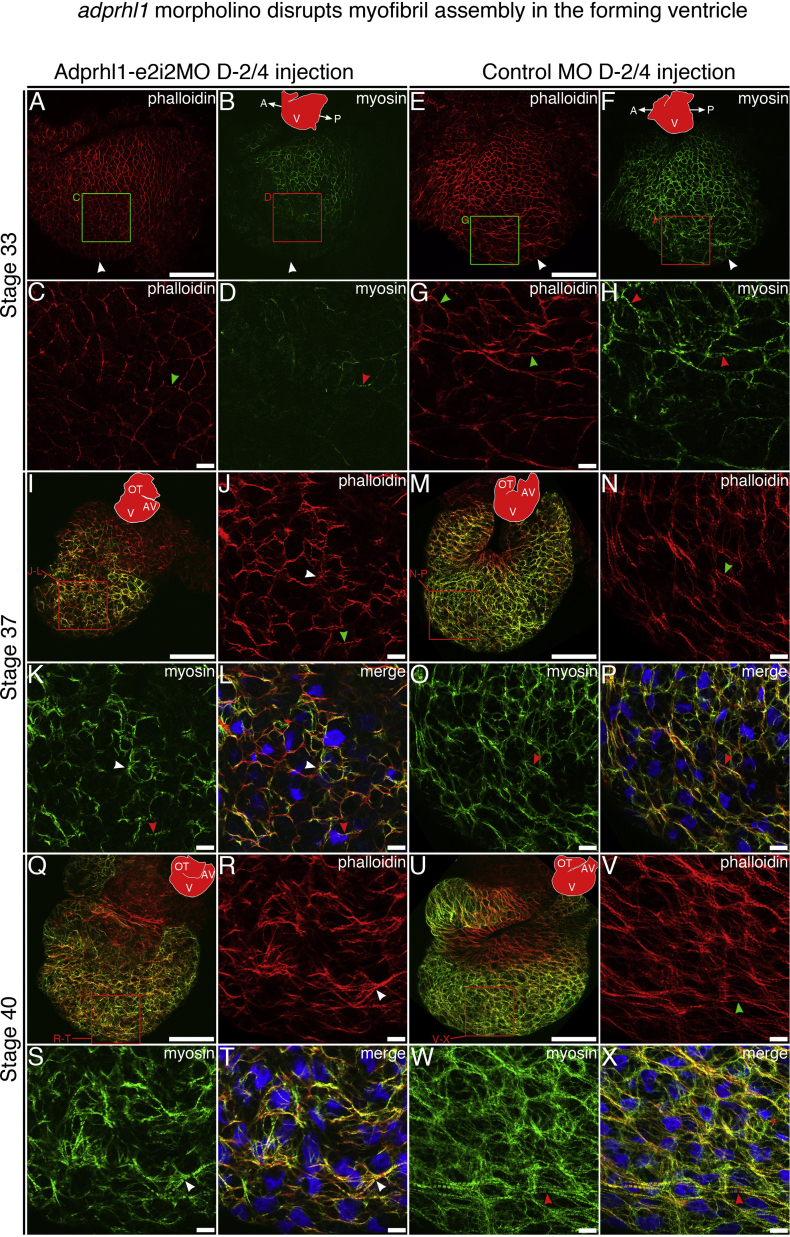
*Adprhl1 morpholino disrupts myofibril assembly in the forming ventricle.* A–D, I–L, Q–T: Dissected hearts from tadpoles injected with Adprhl1-e2i2MO into D-2/4 blastomeres and analyzed at stage 33 (A–D), stage 37 (I–L) and stage 40 (Q–T). E–H, M–P, U–X: Hearts after control morpholino injection into D-2/4 blastomeres. Phalloidin stain of actin filaments (red) and immunocytochemistry for myosin heavy chain (green) are presented. A-H: At the onset of cardiac looping, stage 33, left lateral view of heart tubes (A, B, E, F). Squares denote the position of 105 µm images (C, D, G, H) in the presumptive ventricle region (apex-white arrowheads). Knockdown of *adprhl1* prevents the rearrangement of cortical actin and delays myosin production in the ventricle, thus inhibiting formation of striated myofibrils (coloured arrowheads). Additionally, elongation of cardiomyocytes located near the apex fails to occur. I–P: During ventricle outgrowth, stage 37 hearts (I, M) oriented with anterior surface upwards, showing myofibril patterns in the ventricle wall (J–L, N–P). Myofibrils that eventually form after *adprhl1* knockdown are disarrayed (white arrowheads) whereas control myofibrils near the apex extend in a perpendicular direction (coloured arrowheads). Q–X: At stage 40, comparable series of hearts (Q, U) and ventricle images (R–T, V–X). Despite increased synthesis, no recovery of myofibril order and direction occurs with the *adprhl1* morpholino. Arrowheads mark equivalent sarcomere positions in the paired panels. Scale bars=100 µm (A, E, I, M, Q, U). Scale bars=10 µm (all others).

**Fig. 7:**
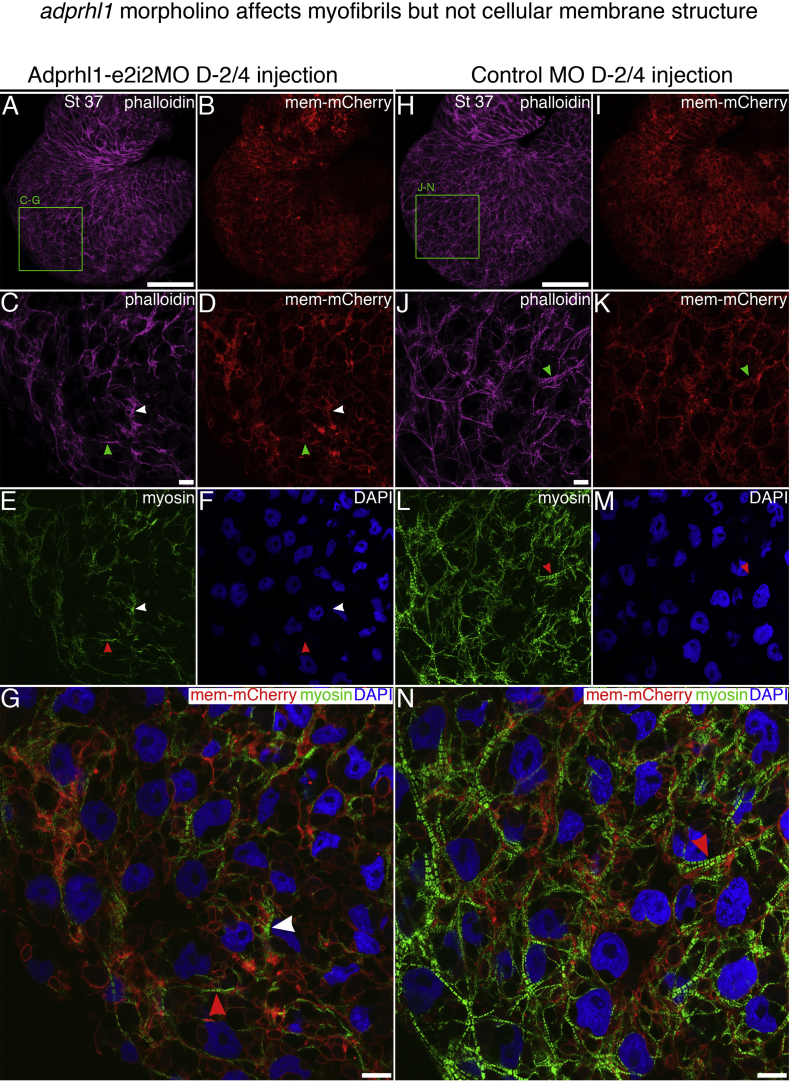
*Adprhl1 morpholino affects myofibrils but not cellular membrane structure.* A–G: A stage 37 heart, dissected after co-injection of Adprhl1-e2i2MO with membrane-tagged lyn-mCherry RNA into D-2/4 blastomeres. H–N: Heart after control morpholino and lyn-mCherry RNA injection into D-2/4 blastomeres. The anterior surface of the hearts (A, B, H, I) lies upwards and magnified 134 µm images (C–G, J–N) show myofibril and membrane patterns in the ventricle wall. Phalloidin actin (magenta-A, C, H, J), membrane-tagged mCherry (red-B, D, I, K), anti-myosin heavy chain (green-E, L), DAPI (F, M) stains, plus RGB-channel merge (G, N). Panels show reduced myofibril content (arrowheads) in the forming ventricle upon *adprhl1* knockdown but no clear change in cellular membrane shape or vesicle structure. Scale bars=100 µm (A, H). Scale bars=10 µm (C–G, J–N). White arrowheads, short, splayed myofibrils in morphant.

**Fig. 3:**
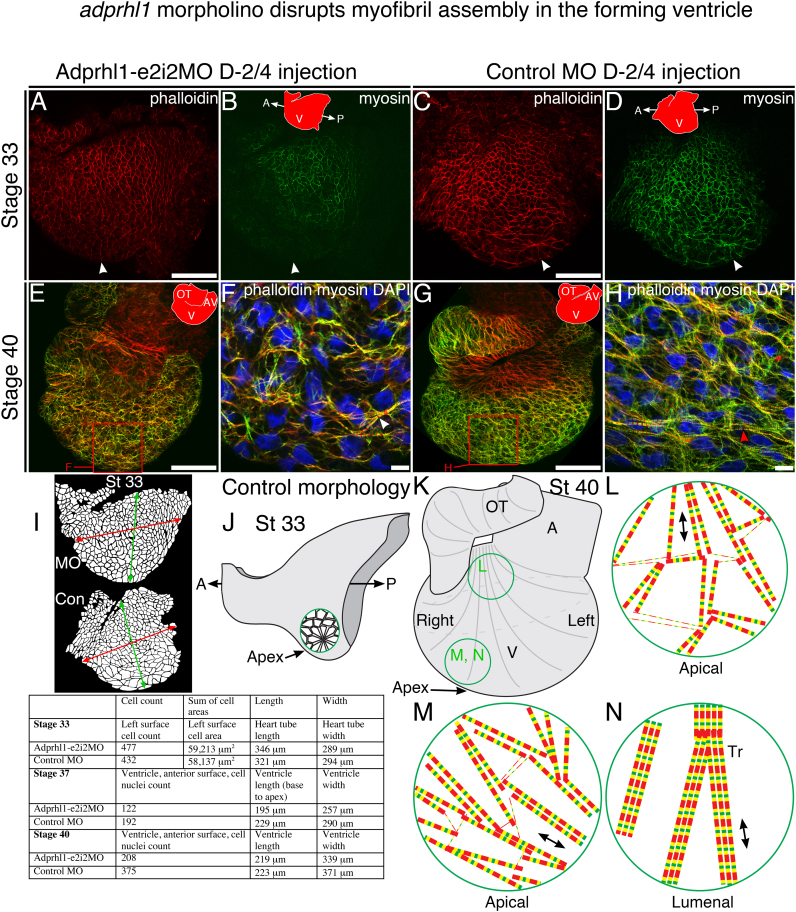
*Adprhl1 morpholino disrupts myofibril assembly in the forming ventricle*. Comparison of dissected hearts from tadpoles injected with Adprhl1-e2i2MO (A, B, E, F) into D-2/4 blastomeres *versus* those containing control morpholino (C, D, G, H). Phalloidin stain of actin filaments (red) and immunocytochemistry for myosin heavy chain (green) is presented (+DAPI nuclei-blue-F, H). S. Fig. 6 contains additional data, including higher magnification images for stage 33, 37 and stage 40 ventricles. A–D: At the onset of cardiac looping, stage 33, left lateral view of heart tubes. (apex-white arrowheads). E–H: After outgrowth of the ventricle, stage 40 hearts (E, G) oriented with anterior surface upwards, showing myofibril patterns (arrowheads) in the ventricle wall (F, H). Scale bars=100 µm (A, C, E, G). Scale bars=10 µm (F, H). I: Cell number and size measurements for the hearts depicted here and S. Fig. 6. At stage 33, cardiac looping is more advanced in the control heart but cell surface area is comparable to the morphant. At stage 37 and 40, fewer cells are present on the surface of smaller morphant ventricles. J–N: Illustrations showing normal morphology and myofibril patterns in the forming ventricle. J: Wildtype stage 33 heart tube, depicting cardiomyocytes on the left side that elongate to form a rosette structure, an early cell movement observed during looping morphogenesis that is absent from morphants. K: A stage 40 heart ventricle, with contours (grey lines) to represent circumference-axes running from base to apex (from inner to outer curvature). Alignment of myofibrils is described relative to these axes. L–N: Simplified arrangement of myofibrils at three locations within the ventricle chamber. Myofibrils near the base (inner curvature-L) orient parallel to the axes. Conversely, around the apex, the predominant orientation of larger myofibrils extends perpendicular to the axes (M), at least for cardiomyocytes on the outer (apical) surface of the chamber. At deeper positions towards the lumen, myofibrils of trabecular (Tr) cardiomyocytes align parallel to the axes (N). There is no abrupt boundary between cardiomyocytes that contain perpendicular or parallel-oriented myofibrils. Moreover, the extent of the perpendicular cells is greater on the anterior ventricle wall (left sided origin) and lesser on the posterior wall (right origin).

**Fig. 4:**
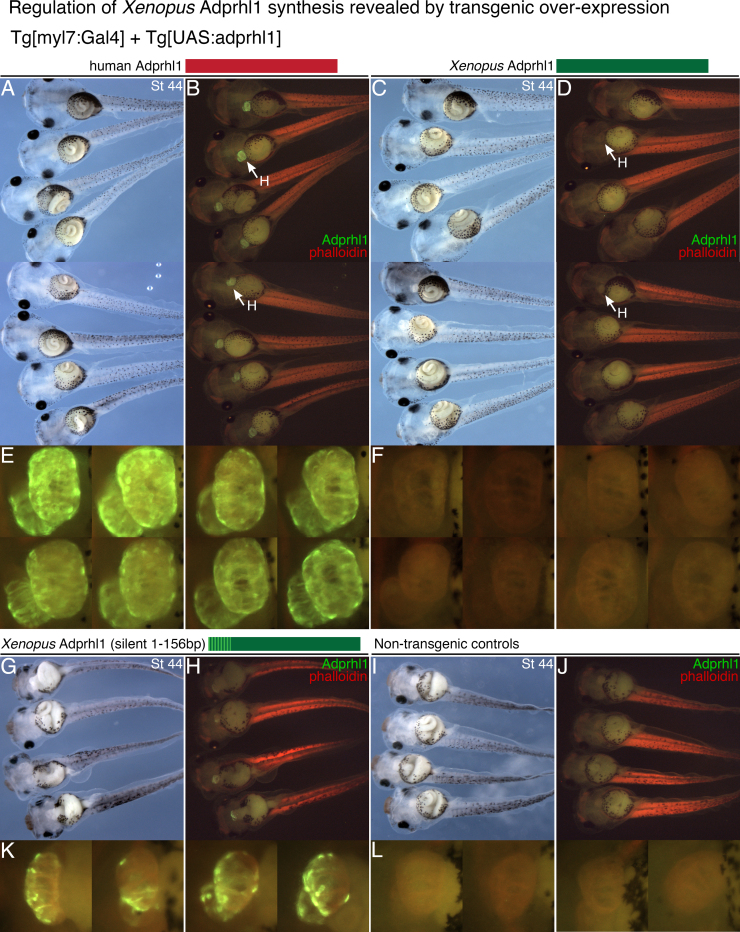
*Regulation of Xenopus Adprhl1 synthesis revealed by transgenic over-expression.* A, B: Eight representative transgenic tadpoles that express human ADPRHL1 protein in their hearts. Each tadpole carries the driver *Tg[myl7:Gal4]* transgene plus a new integration of a *Tg[UAS:human ADPRHL1]* responder transgene. Ventral view of stage 44 tadpoles (A) and matching fluorescence image (B) shows anti-Adprhl1 immunocytochemistry (green) and phalloidin stain in the tail (red). C, D: Conversely, recombinant *Xenopus* Adprhl1 protein does not accumulate in hearts, despite the sibling tadpoles containing the same driver plus a new integration of a *Tg[UAS:Xenopus adprhl1]* responder transgene. E, F: Detail view of the hearts of each tadpole presented. G, H, K: Significantly, the driver plus the *Tg[UAS:Xenopus adprhl1(silent 1-156bp)]* responder containing 36 silent nucleotide changes does produce recombinant *Xenopus* Adprhl1. I, J, L: Non-transgenic control tadpoles that are siblings to those in (G, H, K). The Adprhl1-peptide antibody binds at aa residues 249–266. H, heart.

**Fig. 5:**
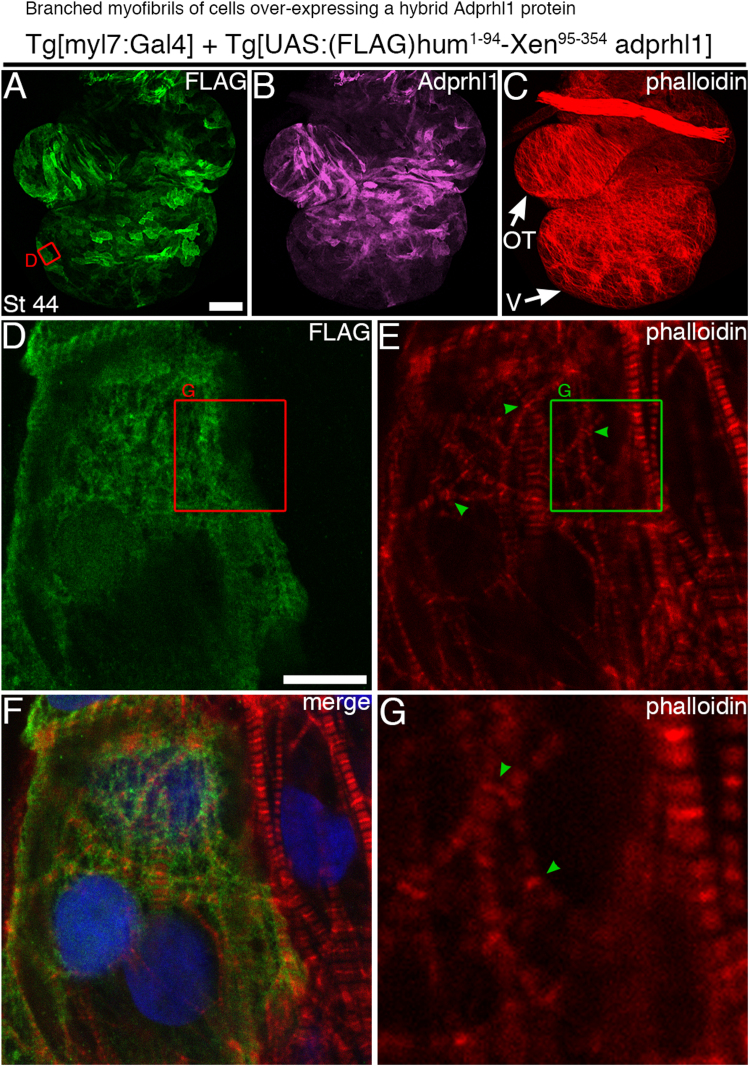
*Branched myofibrils of cells over-expressing a hybrid Adprhl1 protein.* A–C: Expression of a N-terminal human-*Xenopus* hybrid Adprhl1 protein in myocardial cells of a stage 44 transgenic tadpole heart. The tadpole carried the driver *Tg[myl7:Gal4]* transgene plus a new integration of a *Tg[UAS:(FLAG)hum*^*1-94*^*-Xen*^*95-354*^*adprhl1]* responder transgene. The entire dissected heart, anterior view, is presented. A: Anti-FLAG immunocytochemistry. B: Anti-Adprhl1. C: Phalloidin actin stain. V, ventricle; OT, outflow tract. Scale bar=100 µm. D–F: The red square (A) denotes the position of higher magnification images in which cells on the left express recombinant Adprhl1 while those on the right do not. D: Anti-FLAG. E: Phalloidin. F: Channel merge, including DAPI nuclear stain. G: Phalloidin, detail of a 13 µm green square (E) showing actin filament branching (green arrowheads) in cells containing recombinant Adprhl1. Scale bar=10 µm.

**Fig. 6:**
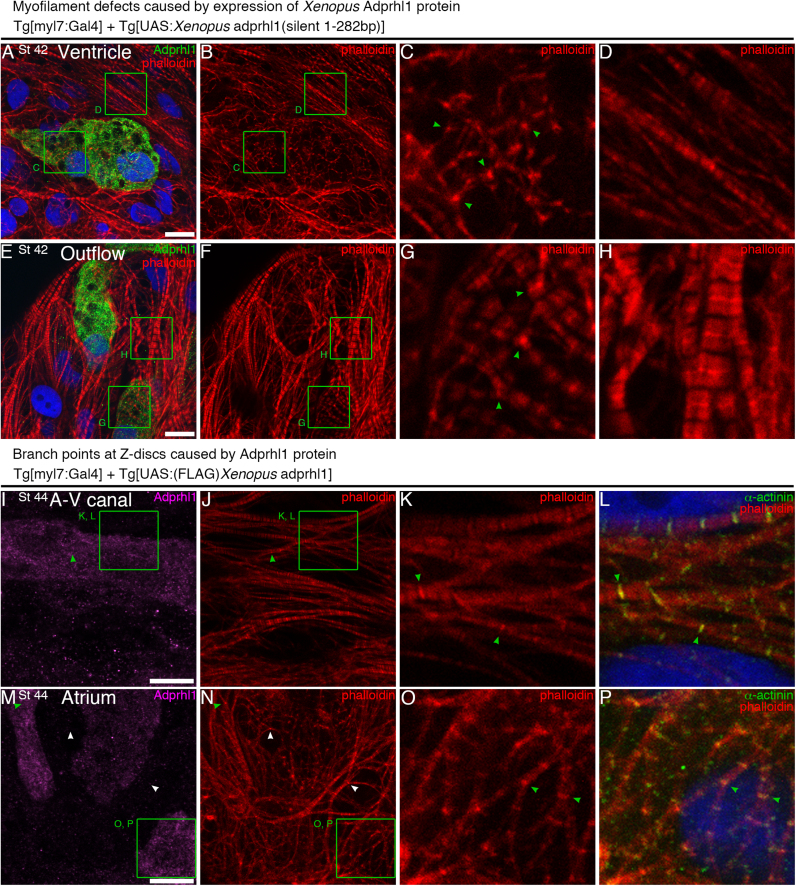
*Myofilament defects caused by expression of Xenopus Adprhl1 protein.* A–D: Rounded cardiomyocyte within the ventricle of a stage 42 heart, carrying *Tg[myl7:Gal4]* driver and *Tg[UAS:Xenopus adprhl1(silent 1-282bp)]* responder transgenes. A: Merge of anti-Adprhl1, phalloidin and DAPI stains. B: Phalloidin alone. C: A 13 µm detail (green square, A, B) showing severe myofilament defects (green arrowheads) in the Adprhl1-positive cell. D: Detail of myofibrils in an adjacent, control cardiomyocyte. E–H: *Xenopus* Adprhl1 in cells of the proximal outflow tract. G: Phalloidin stain of branched filaments and circular foci (green arrowheads) in an Adprhl1-positive cell. H: Myofibrils in an adjacent cell. I–L: Branched myofibrils (green arrowheads) within the atrio-ventricular canal of a stage 44 heart with the driver plus *Tg[UAS:(FLAG)Xenopus adprhl1]* transgenes. I: Anti-Adprhl1. J: Phalloidin. K: 13 µm detail of phalloidin stain. L: Merge of phalloidin, anti-α-actinin and DAPI. M-P: FLAG-*Xenopus* Adprhl1 production in the forming atria. Actin branch points are detected in areas of low Adprhl1 concentration (white arrowheads) but are prevalent in cells with excess recombinant Adprhl1 (green arrowheads). The branches occur at Z-discs, labelled by α-actinin. Scale bars=10 µm.

**Fig. 7:**
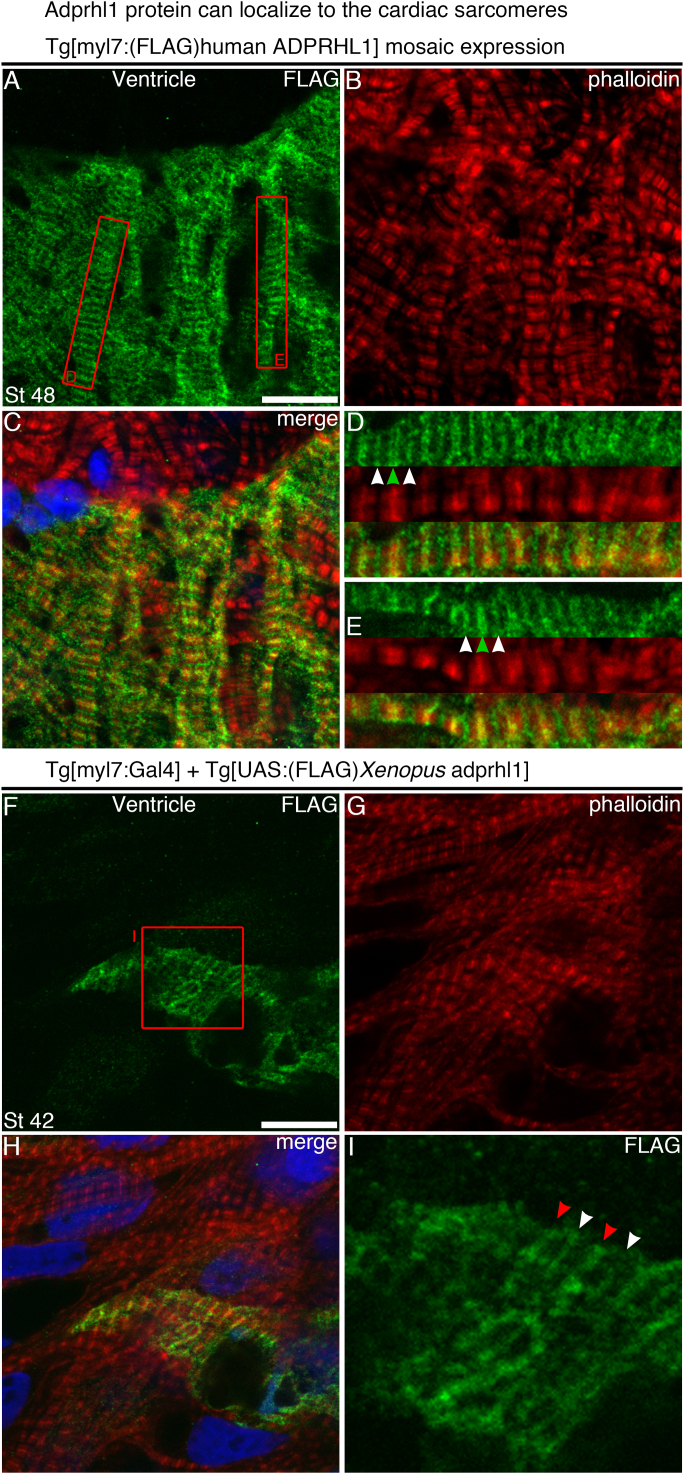
*Adprhl1 protein can localize to the cardiac sarcomeres.* A–E: Expression of human ADPRHL1 protein within a cluster of ventricular cells from a stage 48 *Tg[myl7:(FLAG)human ADPRHL1]* transgenic tadpole. A: Anti-FLAG. B: Phalloidin actin stain. C: Channel merge, including DAPI. D, E: Red rectangles (A) denote the position of 22 µm lengths of individual myofibrils. Human ADPRHL1 localizes to two stripes either side of the Z-disc (green arrowheads) and to the H-zone (white arrowheads). F–I: Similar localization of FLAG-*Xenopus* Adprhl1 within a cell of a stage 42 *Tg[myl7:Gal4]* plus *Tg[UAS:(FLAG)Xenopus adprhl1]* ventricle. F: Anti-FLAG. G: Phalloidin. H: Channel merge. I: Anti-FLAG, detail of the 13 µm red square (F). Z-disc (red arrowheads). Scale bars=10 µm.

**Movie 1:**
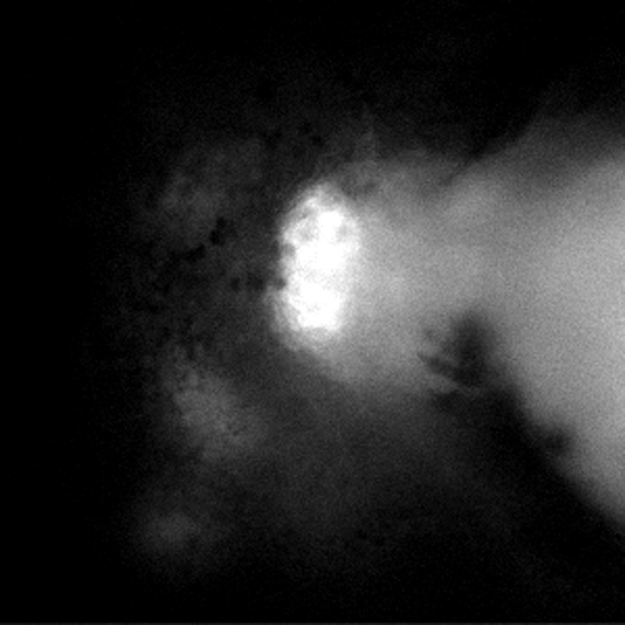
*Movies of the adprhl1 morpholino heart beat defect*. Time-lapse movies captured at 300 ms/frame, ventral views of *Tg[actc1:GFP]* heart fluorescence of stage 41 tadpoles after injection with Adprhl1-e2i2MO. The first tadpole depicted is a non-injected, stage-matched sibling control. The second tadpole was injected with morpholino into D-2/4 blastomeres and has a completely inert heart. The atrial region is mostly obscured by the ventricle and does exhibit some pulsing movement. The third tadpole also contains D-2/4-morpholino and has a severe, though partial, heart beat defect. The atria initiate a contraction that is weakly propagated through the ventricle. The overall appearance of these tadpoles is presented in S. Fig. 3(I–Q). Anterior is to the left. A video clip is available online.

**Movie 2:**
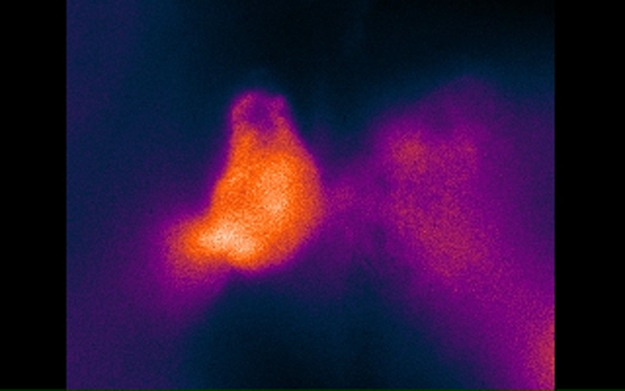
*Calcium imaging inert hearts produced by adprhl1 morpholino*. Time-lapse movie of a stage 42 tadpole that was co-injected with Adprhl1-e2i2MO and also R-GECO1 RNA into D-2/4 blastomeres. The injection masses were 20 ng morpholino and 800 pg RNA. Ventral view of the cardiac ventricle is displayed, anterior to left. The small tubular heart of this tadpole is mechanically inert. Nonetheless, the red fluorescent calcium indicator protein, R-GECO1, detects an active intracellular calcium wave. The wave initiates at the inflow region, which is positioned more dorsally than the ventricle so appears fainter (immediately to the right of the ventricle in the movie). It then propagates through the ventricular portion of the looped tube (from top to bottom of movie) and outflow tract (left) without apparent obstruction. Greyscale images were captured and false-coloured in Image J using the GEM LUT. Movies are 10 s duration, captured at 20 frames per second and are played back at 5 fps.

**Movie 3:**
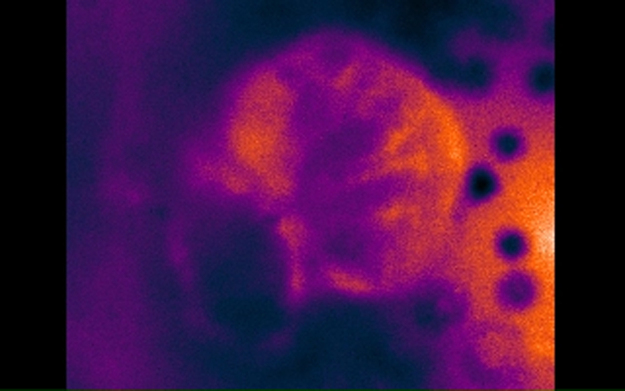
*Calcium imaging a control heart*. Companion to S. Movie 2. Movie shows a sibling tadpole injected with R-GECO1 RNA only. In this normal heart, the increase in Ca^2+^-mediated fluorescence occurs as ventricular systole commences. The wave propagates from the left wall of the ventricular chamber towards the right, which is comparable to the posterior to anterior wave of a looping stage tubular heart.

## Appendix A Supplementary material

Fig. 1 *Expression of Adprhl1 in mouse embryos and its dependency on Nkx2-5.* A–D: Myocardial expression of murine *Adprhl1* mRNA after sagittal section *in situ* hybridization at E11.5 and E14.5 stages of development. Heart regions are magnified (B, D). Anterior is to top. E–H: For comparison, expression of the cardiac transcription factor, *Nkx2-5,* at the same stages. Scale bars=1 mm (A, E), 500 µm (C, G). H, heart; pH, pharynx domain; S, spleen; To, tongue; A.Mc, atrial myocardium; V.Mc, ventricular myocardium; LA, left atrium; RV, right ventricle; IVS, interventricular septum wall. I: RT-PCR amplification of *Adprhl1* and other representative cardiac cDNAs. At E9.0, wildtype (WT) and *Nkx2-5*-null (Null, *Nkx2-5*^*gfp/gfp*^) sibling embryos remain morphologically similar. Nonetheless, *Adprhl1* expression appears sensitive to loss of *Nkx2-5*, along with four other well-characterized transcripts. Ensembl (genome GRCm38) transcript nomenclature is used. J: Western blot detection of ADPRHL1 protein within individual E11.5 mouse hearts. Three ADPRHL1 protein bands are identified by the antibody; the signal for the 35 kDa. protein appears stronger than for the 25 and 23 kDa. species and so different exposures are presented. Moreover, the abundance of the 35 kDa. ADPRHL1 protein is reduced in a heart that contains only 25% of the normal activity of *Nkx2-5*. WT, wildtype control; Het, heterozygote (*Nkx2-5*^*+/gfp*^); Hyp, hypomorphic mutant (*Nkx2–5*^*gfp/IRES-cre*^). Comparing amino acid sequences, murine ADPRHL1 should be smaller than the human and *Xenopus* proteins, although it is unclear quite why it resolves at 35 kDa instead of 39 kDa. The NKX2-5 protein, 60 kDa (presumed sumoylated) and 34 kDa bands are also shown and the samples are normalized with myosin heavy chain (MyHC).

.

Fig. 2 *Adprhl1 morpholino sequences and activity.* A: Sequences of morpholino oligonucleotides that target *X. laevis adprhl1* RNA splicing aligned with the corresponding exon 2-intron 2, and intron 2-exon 3 boundaries. Intron sequences are lower case. Mismatched control morpholinos have sequence changes highlighted (blue). In the outbred animals used in the study, two variants of *X. laevis adprhl1* intron 2 were identified with sequence differences near the intron 2-exon 3 boundary shown (green)(Accession GU188989, GU188990). The inbred J-line strain, JGI genome 7.2 sequence of *adprhl1* (scaffold 167628) and the homeolog (scaffold 338390) are also depicted. No ESTs exist that map to the second locus. Nonetheless, the homeolog cDNA was identified in our experiments and thus was considered when designing morpholinos and PCR primers. B: RT-PCR of *X. laevis adprhl1* cDNA amplified from four individual stage 30 embryos that had been injected with morpholinos into D-2/4 blastomeres. The Adprhl1-e2i2MO causes retention of intron 2 within the mRNA, introducing a premature stop codon and potentially a 128 amino acid product (instead of full length 354 aa). The Adprhl1-i2e3MO causes deletion of exon 3 from the mRNA, with a loss of 126 bases (42 amino acids). PCR primers p1855 and p1856 were used. **C:** RT-PCR of *adprhl1* cDNA from dissected stage 42 hearts. Primers distributed across the coding sequence each produce a single product, suggesting that in *Xenopus* embryos at least, there are no transcript variants with altered exon composition.

.

Fig. 12 *Binary transgene system for cardiac adprhl1 over-expression.* Reproducible cardiac expression of transgenes in founder generation transgenic tadpoles can be achieved using the Gal4/UAS binary system. A: Drawing of transgene DNAs used in the *adprhl1* over-expression study. B: Experimental scheme, indicating the stages of development that transgenic tadpoles were analyzed. C: Identification of transgenic tadpoles based on their eye fluorescence. A stage 44 tadpole that carries both the driver *Tg[myl7:Gal4, γCrys:eCFP]* transgene plus a new integration of a *Tg[UAS:Xenopus adprhl1, γCrys:DsRed1]* responder transgene. In this double transgenic tadpole, both left and right eyes have widespread cyan and red fluorescence. D: A full list of the transgenes used for over-expression of Adprhl1 proteins, with a summary of their activity and sequence differences compared to *Xenopus adprhl1*. E: Sequences of the first 282 bp of *adprhl1* coding cDNA, for *Xenopus* and human, plus hybrid (1-52aa) and silent (synonymous) mutation transgenes. Nucleotides that are changed from the *Xenopus* sequence and present in the human ortholog are coloured red, additional silent changes are green.

## Footnotes

Appendix A Supplementary data associated with this article can be found in the online version at 10.1016/j.ydbio.2016.05.006.
