# Supplementary material for: The cardiac-restricted protein ADP-ribosylhydrolase-like 1 is essential for heart chamber outgrowth and acts on muscle actin filament assembly
Source: Dev Biol. 2016 Aug 15;416(2):373–88. doi: 10.1016/j.ydbio.2016.05.006 (PMC4990356; doi:10.1016/j.ydbio.2016.05.006)

Supplementary Figure 1

Expression of *Adprhl1* in mouse embryos and its dependency on *Nkx2-5*

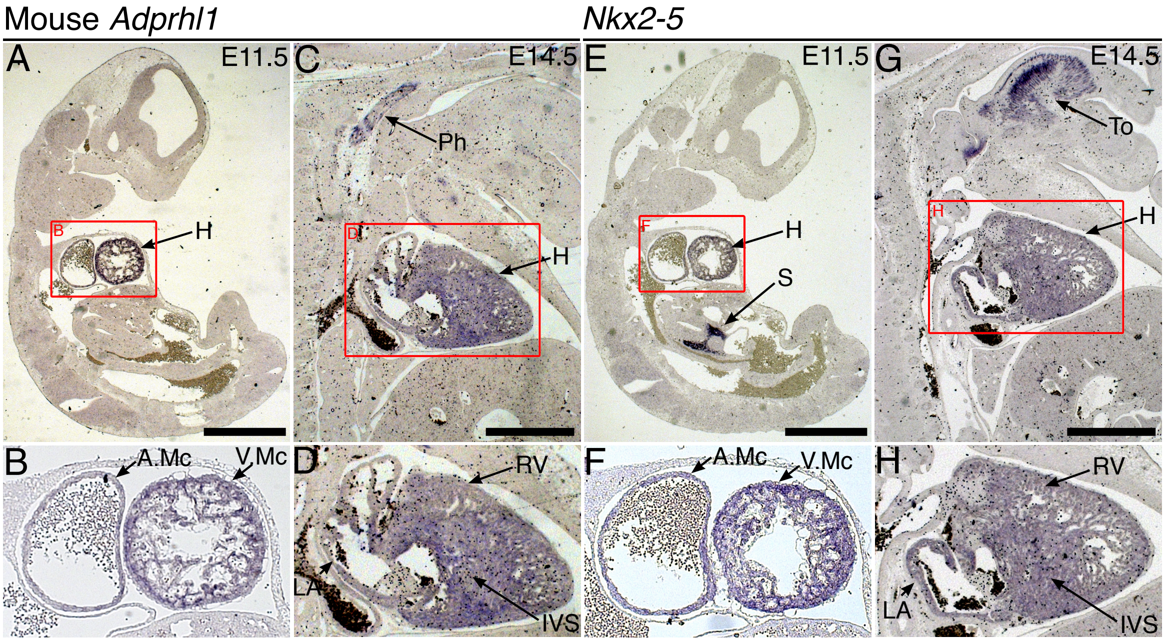

I RT-PCR of *Adprhl1* at E9.0 in wildtype and *Nkx2-5*-null embryos

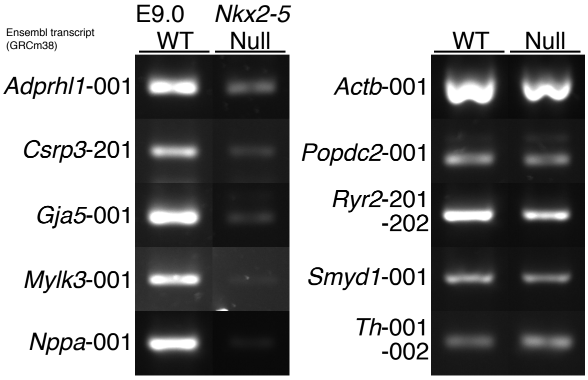

Gene - transcript	Sense Primer	Antisense Primer
Adprhl1-001	CCATCCGGCACATGGCAGAGTAC	GGCGATCGTCCCTGTCGCTC
Csrp3-201	TGAGCCGGCAGAGACCACACA	GAAGGGTTGCTTGTGGTGGCT
Gja5-001	GAAGAAGCCAACTCCAGGGA	GATGATCTGCAGTACCCAAT
Mylk3-001	ATGTCAGGAGTTTCAGAGGA	CGATTGCTACGGACAATTCCA
Nppa-001	TTCTCCATCACCCTGGGCTTC	AGAGCCCTCAGTTTGCTTTTC
Actb-001	GTGGGCCGCCCTAGGCACCAG	CTCTTTGATGTCACGCACGATTTC
Popdc2-001	CCTCTCAGGCCGGGTTCGAGT	GTCTAGAGGCCAGGTTGCCGCTG
Ryr2-201, -202	CACGTCTGCATGTGCAGTTCC	GGCCTATGCCTGGGAGGCCAT
Smyd1-001	ACAATAGGCAGCATGGAGAAC	CATGGATTTCACTGCCTCATG
Th-001, -002	CATGTTGGCTGACCGCACATT	ACGAAGTACACCGGCTGGTAG

J Western blot of ADPRHL1 protein at E11.5 in wildtype and *Nkx2-5*-hypomorph hearts

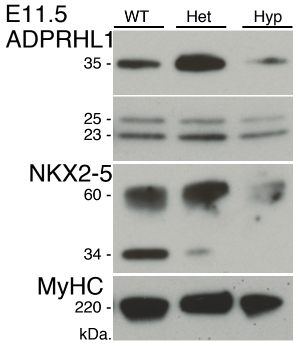

Supplement: Fig. 1 — Expression of Adprhl1 in mouse embryos and its dependency on Nkx2-5. A–D: Myocardial expression of murine Adprhl1 mRNA after sagittal section in situ hybridization at E11.5 and E14.5 stages of development. Heart regions are magnified (B, D). Anterior is to top. E–H: For comparison, expression of the cardiac transcription factor, Nkx2-5, at the same stages. Scale bars=1 mm (A, E), 500 µm (C, G). H, heart; pH, pharynx domain; S, spleen; To, tongue; A.Mc, atrial myocardium; V.Mc, ventricular myocardium; LA, left atrium; RV, right ventricle; IVS, interventricular septum wall. I: RT-PCR amplification of Adprhl1 and other representative cardiac cDNAs. At E9.0, wildtype (WT) and Nkx2-5-null (Null, Nkx2-5gfp/gfp) sibling embryos remain morphologically similar. Nonetheless, Adprhl1 expression appears sensitive to loss of Nkx2-5, along with four other well-characterized transcripts. Ensembl (genome GRCm38) transcript nomenclature is used. J: Western blot detection of ADPRHL1 protein within individual E11.5 mouse hearts. Three ADPRHL1 protein bands are identified by the antibody; the signal for the 35 kDa. protein appears stronger than for the 25 and 23 kDa. species and so different exposures are presented. Moreover, the abundance of the 35 kDa. ADPRHL1 protein is reduced in a heart that contains only 25% of the normal activity of Nkx2-5. WT, wildtype control; Het, heterozygote (Nkx2-5+/gfp); Hyp, hypomorphic mutant (Nkx2–5gfp/IRES-cre). Comparing amino acid sequences, murine ADPRHL1 should be smaller than the human and Xenopus proteins, although it is unclear quite why it resolves at 35 kDa instead of 39 kDa. The NKX2-5 protein, 60 kDa (presumed sumoylated) and 34 kDa bands are also shown and the samples are normalized with myosin heavy chain (MyHC). [file mmc1.pdf]
