# Supplementary material for: The cardiac-restricted protein ADP-ribosylhydrolase-like 1 is essential for heart chamber outgrowth and acts on muscle actin filament assembly
Source: Dev Biol. 2016 Aug 15;416(2):373–88. doi: 10.1016/j.ydbio.2016.05.006 (PMC4990356; doi:10.1016/j.ydbio.2016.05.006)
Supplement: Fig. 2 — Adprhl1 morpholino sequences and activity. A: Sequences of morpholino oligonucleotides that target X. laevis adprhl1 RNA splicing aligned with the corresponding exon 2-intron 2, and intron 2-exon 3 boundaries. Intron sequences are lower case. Mismatched control morpholinos have sequence changes highlighted (blue). In the outbred animals used in the study, two variants of X. laevis adprhl1 intron 2 were identified with sequence differences near the intron 2-exon 3 boundary shown (green)(Accession GU188989, GU188990). The inbred J-line strain, JGI genome 7.2 sequence of adprhl1 (scaffold 167628) and the homeolog (scaffold 338390) are also depicted. No ESTs exist that map to the second locus. Nonetheless, the homeolog cDNA was identified in our experiments and thus was considered when designing morpholinos and PCR primers. B: RT-PCR of X. laevis adprhl1 cDNA amplified from four individual stage 30 embryos that had been injected with morpholinos into D-2/4 blastomeres. The Adprhl1-e2i2MO causes retention of intron 2 within the mRNA, introducing a premature stop codon and potentially a 128 amino acid product (instead of full length 354 aa). The Adprhl1-i2e3MO causes deletion of exon 3 from the mRNA, with a loss of 126 bases (42 amino acids). PCR primers p1855 and p1856 were used. C: RT-PCR of adprhl1 cDNA from dissected stage 42 hearts. Primers distributed across the coding sequence each produce a single product, suggesting that in Xenopus embryos at least, there are no transcript variants with altered exon composition. [file mmc2.pdf]

Supplementary Figure 2

Xenopus adprhl1 morpholino sequences and activity

A *X. laevis adprhl1* RNA splice interfering morpholino sequences

Exon 2 - intron 2 boundary

```
GU188989 +
GU188990        5’-AATGAAAAAGgtttgtaagatgctgagcctggaaacctatagattctggcaaaaggctgc
JGI 7.2 scaffold 167628    [AAAAGgtttgtaaggtgctgagccc]
JGI 7.2 scaffold 338390    [AAAAGgtttggaagatgctaagcct]
                        3’-TTTTCCAAACATTCTACGACTCGGA-5’——— Adprhl1-e2i2MO
                        3’-TTTTACAAAAATTATACGAATCGAA-5’——— Adprhl1-e2i2MOMis
```

Intron 2 - exon 3 boundary

```
GU188989        5’-caaagagggttctttagggaagacaaaagtgactctagttgtttcttagGTTCAGGATT
GU188990        5’-caaagagggttctttagggaagacaaccagtgactctagttgtttcttagGTTCAGGATT
JGI 7.2 scaffold 167628                          [cagtgactttagtcgtttcttagGT]
JGI 7.2 scaffold 338390                          [cagtgactctagttgtttcttagGT]
   Adprhl1-i2e3MO ———                          3’-GTCACTGAGATCAACAAAGAATCCA-5’
Adprhl1-i2e3MOMis ———                          3’-GTAACTAAGATAAACAAAAAATCAA-5’
```

B *X. laevis adprhl1* morpholino RT-PCR

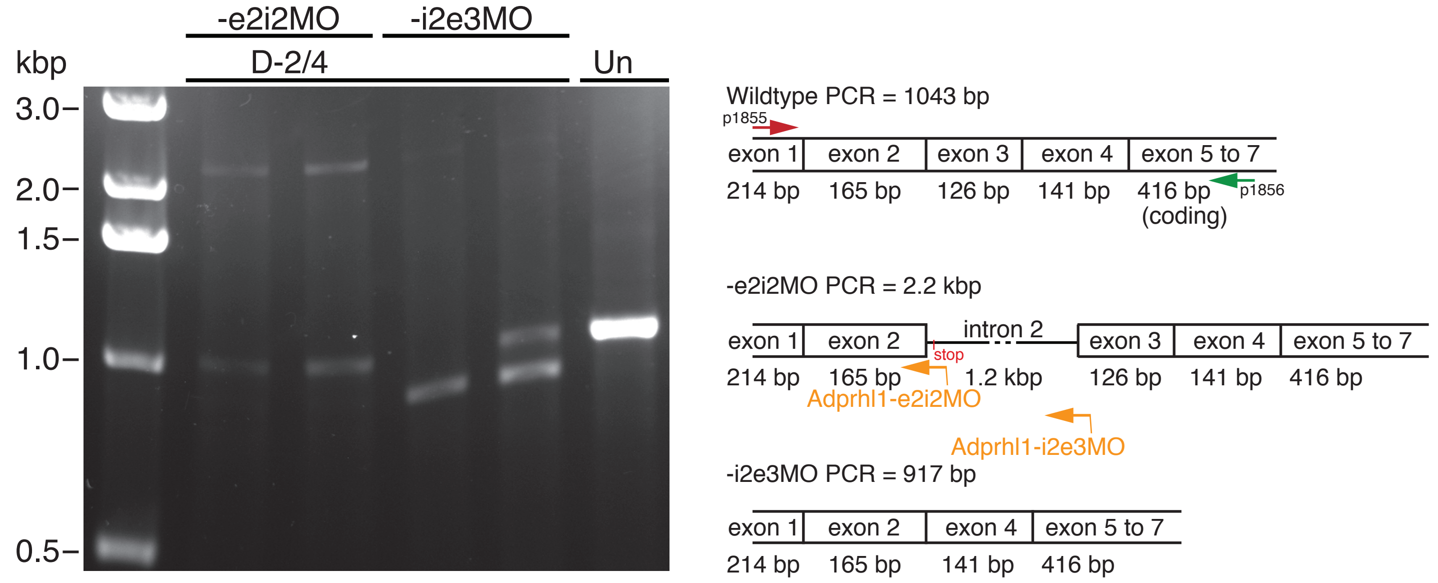

C *X. laevis* wild type stage 42 heart *adprhl1* RT-PCRs

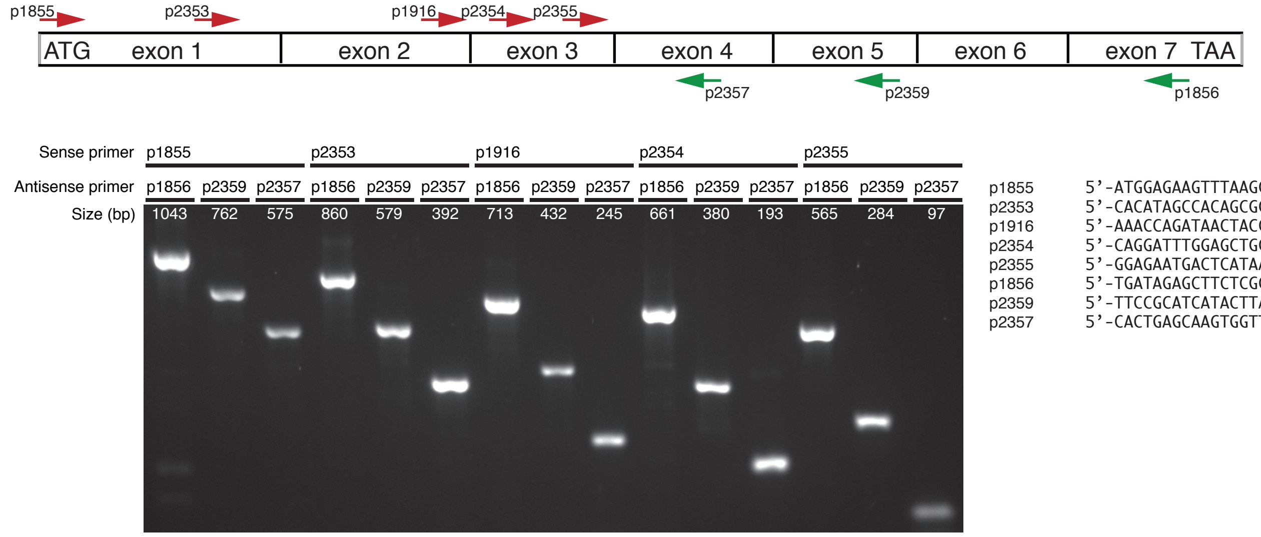

p1855	5’-ATGGAGAAGTTTAAGGCTGC-3’
p2353	5’-CACATAGCCACAGCGGAATC-3’
p1916	5’-AAACCAGATAACTACCTGCT-3’
p2354	5’-CAGGATTTGGAGCTGCAACA-3’
p2355	5’-GGAGAATGACTCATAACCATC-3’
p1856	5’-TGATAGAGCTTCTCGCCAAGG-3’
p2359	5’-TTCCGCATCATACTTATCAGG-3’
p2357	5’-CACTGAGCAAGTGGTTTCTC-3’
