# Supplementary material for: The cardiac-restricted protein ADP-ribosylhydrolase-like 1 is essential for heart chamber outgrowth and acts on muscle actin filament assembly
Source: Dev Biol. 2016 Aug 15;416(2):373–88. doi: 10.1016/j.ydbio.2016.05.006 (PMC4990356; doi:10.1016/j.ydbio.2016.05.006)
Supplement: Fig. 12 — Binary transgene system for cardiac adprhl1 over-expression. Reproducible cardiac expression of transgenes in founder generation transgenic tadpoles can be achieved using the Gal4/UAS binary system. A: Drawing of transgene DNAs used in the adprhl1 over-expression study. B: Experimental scheme, indicating the stages of development that transgenic tadpoles were analyzed. C: Identification of transgenic tadpoles based on their eye fluorescence. A stage 44 tadpole that carries both the driver Tg[myl7:Gal4, γCrys:eCFP] transgene plus a new integration of a Tg[UAS:Xenopus adprhl1, γCrys:DsRed1] responder transgene. In this double transgenic tadpole, both left and right eyes have widespread cyan and red fluorescence. D: A full list of the transgenes used for over-expression of Adprhl1 proteins, with a summary of their activity and sequence differences compared to Xenopus adprhl1. E: Sequences of the first 282 bp of adprhl1 coding cDNA, for Xenopus and human, plus hybrid (1-52aa) and silent (synonymous) mutation transgenes. Nucleotides that are changed from the Xenopus sequence and present in the human ortholog are coloured red, additional silent changes are green. [file mmc12.pdf]

Supplementary Figure 12

Binary transgene system for cardiac *adprhl1 over*-expression.

A Transgene diagrams

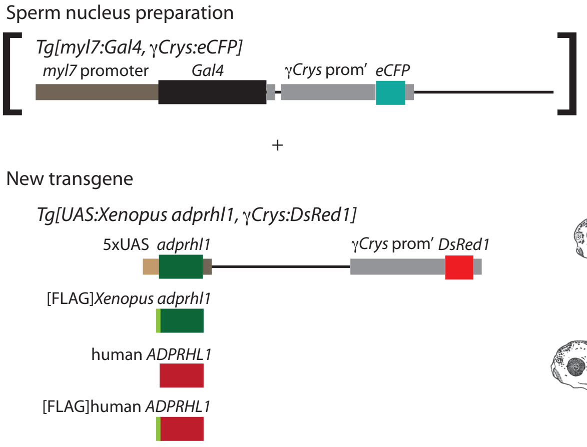

B Produce founder generation transgenic tadpoles

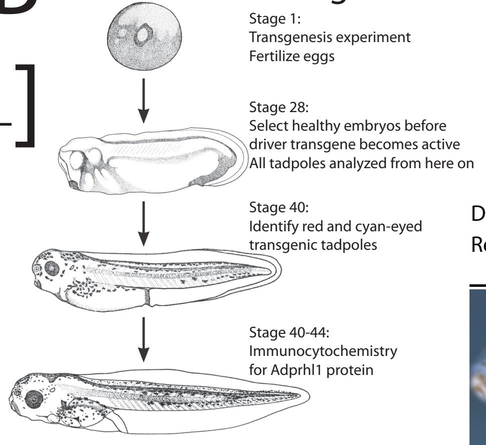

C Identification of transgenic tadpoles

Driver: *Tg[myl7:Gal4, γCrys:eCFP]*

Responder: *Tg[UAS:Xenopus adprhl1, γCrys:DsRed1]*

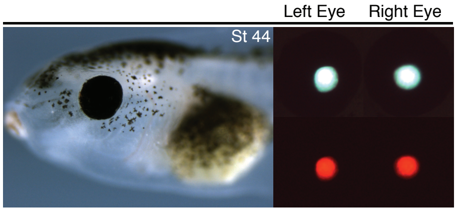

D

Transgene list<br>*Adprhl1* transgenes:	Recombinant Adprhl1 protein signal?	*Adprhl1* coding cDNA sequence exchanged	Number of nucleotide changes (from *Xenopus*)	Number of amino acid changes (from *Xenopus*)	Number of stable transgenic lines analyzed
*Tg[UAS:**Xenopus adprhl1**, γCrys:DsRed1]*	None				3
*Tg[UAS:**human ADPRHL1**, γCrys-DsRed1]*	Strong	1-1062 bp	320	90	5
*Tg[UAS:**(FLAG)Xenopus adprhl1**, γCrys:DsRed1]*	None				4
*Tg[UAS:**(FLAG)human ADPRHL1**, γCrys:DsRed1]*	Strong	1-1062 bp	320	90	2
*Tg[myl7:**Xenopus adprhl1**, γCrys:DsRed1]*	None				
*Tg[myl7:**(FLAG)human ADPRHL1**, γCrys:DsRed1]*	Strong	1-1062 bp	320	90	
Human-*Xenopus* hybrid *adprhl1* transgenes:<br>(Numbers refer to protein sequence)<br>N-terminal switch:					
*Tg[UAS:**(FLAG)hum$^{1-94}$-Xen$^{95-354}$ adprhl1**, γCrys:DsRed1]*	Strong	1-282 bp	100	36	
*Tg[UAS:**(FLAG)Xen$^{1-94}$-hum$^{95-354}$ adprhl1**, γCrys:DsRed1]*	Weak	277-1062 bp	221	54	
C-terminal switch:					
*Tg[UAS:**(FLAG)hum$^{1-265}$-Xen$^{266-354}$ adprhl1**, γCrys:DsRed1]*	Strong	1-794 bp	230	68	
*Tg[UAS:**(FLAG)Xen$^{1-265}$-hum$^{266-354}$ adprhl1**, γCrys:DsRed1]*	None	795-1062 bp	90	22	
N-terminal, small peptide switches:					
*Tg[UAS:**hum$^{1-52}$-Xen$^{53-354}$ adprhl1**, γCrys:DsRed1]*	Medium	1-156 bp	63	21	2
*Tg[UAS:**Xen$^{1-52}$-hum$^{53-94}$-Xen$^{95-354}$ adprhl1**, γCrys:DsRed1]*	None	163-282 bp	36	15	
*TgUAS:**hum$^{1-19}$-Xen$^{20-31}$-hum$^{32-52}$-Xen$^{53-354}$ adprhl1**, - γCrys:DsRed1]*	Weak	1-57 + 94-156 bp	42	11	
*Tg[UAS:**Xen$^{1-19}$-hum$^{20-31}$-Xen$^{32-354}$ adprhl1**, γCrys:DsRed1]*	None	58-93 bp	21	10	
Silent *adprhl1* cDNA nucleotide changes:<br>(Numbers refer to coding cDNA sequence)					
*Tg[UAS:Xenopus adprhl1**(silent 1-156bp)**, γCrys:DsRed1]*	Medium	1-156 bp	36	0	
*Tg[UAS:Xenopus adprhl1**(silent 1-282bp)**, γCrys:DsRed1]*	Medium	1-282 bp	69	0	2

E Sequences of 5′-coding *adprhl1* cDNAs (1-282 bp)

Xenopus adprhl1
1-100 ATGGAGAAGTTTAAGGCTGCAATGCTTCTAGCTGGGACAGGGGATGCTTTGGGCTATAAGAACTTTAGCTGGGTATTTTGTGCATCAGGCGTAAAAATCC
101-200 AAGAAGAGCTAAAACAACTTGGGGGATTGGAAAATTTGGTTCTGTCCATAGACGGCTGGCCAGTGAGTAACAATACTCTAATGCACATAGCCACAGCGGA
201-282 ATCACTAGTCTCAGATTACTGGAGCATAGAAGACCTGTACCGTGACATGGTAAAACGTTACATTGATGTTGTTGACAAGCTG

Human *ADPRHL1*
1-100 ATGGAGAAATTTAAGGCTGCGATGTTGCTGGGGAGCGTCGGCGATGCTCTTGGCTACAGAAATGTCTGCAAGGAGAACAGCACTGTAGGCATGAAGATCC
101-200 AGGAGGAGCTGCAACGTTCCGGGGGCCTGGACCACCTCGTACTCTCGCCAGGAGAATGGCCCGTGAGTGACAACACCATCATGCACATCGCAACCGCCGA
201-282 GGCCCTCACCACAGACTACTGGTGCCTGGATGATCTGTACCGGGAGATGGTGAGATGCTATGTGGAAATCGTTGAGAAGCTT

Hum$^{1-52}$-*Xen*$^{53-354}$ *adprhl1*
1-100 ATGGAGAAATTTAAGGCTGCGATGTTGCTGGGGAGCGTCGGCGATGCTCTTGGCTACAGAAATGTCTGCAAGGAGAACAGCACTGTAGGCATGAAGATCC
101-200 AGGAGGAGCTGCAACGTTCCGGGGGCCTGGACCACCTCGTACTCTCGCCAGGAGAATGGCCAGTGAGTAACAATACTCTAATGCACATAGCCACAGCGGA
201-282 ATCACTAGTCTCAGATTACTGGAGCATAGAAGACCTGTACCGTGACATGGTAAAACGTTACATTGATGTTGTTGACAAGCTG

Xenopus adprhl1(silent1-156bp)
1-100 ATGGAGAAATTTAAGGCTGCGATGTTGCTGGCGGGCACCGGCGATGCTCTTGGCTACAAAAATTTTAGTTGGGTGTTTTGCGCTTCTGGAGTGAAGATCC
101-200 AGGAGGAGCTGAAGCAGCTCGGGGGCCTGGAAAACCTCGTACTCTCGATAGACGGCTGGCCAGTGAGTAACAATACTCTAATGCACATAGCCACAGCGGA
201-282 ATCACTAGTCTCAGATTACTGGAGCATAGAAGACCTGTACCGTGACATGGTAAAACGTTACATTGATGTTGTTGACAAGCTG

Xenopus adprhl1(silent1-282bp)
1-100 ATGGAGAAATTTAAGGCTGCGATGTTGCTGGCGGGCACCGGCGATGCTCTTGGCTACAAAAATTTTAGTTGGGTGTTTTGCGCTTCTGGAGTGAAGATCC
101-200 AGGAGGAGCTGAAGCAGCTCGGGGGCCTGGAAAACCTCGTACTCTCGATCGATGGATGGCCCGTGAGTAATAACACCCTGATGCACATCGCAACCGCCGA
201-282 GTCTCTCGTGTCTGACTACTGGAGTATCGAGGATCTGTACCGGGATATGGTGAAGCGCTATATCGACGTGGTTGATAAGCTT
