# Supplementary material for: The cardiac-restricted protein ADP-ribosylhydrolase-like 1 is essential for heart chamber outgrowth and acts on muscle actin filament assembly
Source: Dev Biol. 2016 Aug 15;416(2):373–88. doi: 10.1016/j.ydbio.2016.05.006 (PMC4990356; doi:10.1016/j.ydbio.2016.05.006)
Supplement: Supplementary file 4 — Supplementary material [file mmc20.docx]

# Supplementary Tables

**Supplementary Table I.** *Number of cardiac-Gal4/UAS transgenic tadpoles in which recombinant Adprhl1 protein was detected (founder generation animals).*

|  |  | Recombinant Adprhl1 detection | | | |
| --- | --- | --- | --- | --- | --- |
| Transgenes:  Tg[myl7:Gal4]  + Tg[UAS:adprhl1] | Total number | Uniform positive signal | Small clone of positive cells | One positive cell | Negative |
| Human: | | | | | |
| Human ADPRHL1 | 26 | 23 | 1 | 0 | 2 |
| (FLAG)human ADPRHL1 | 40 | 35 | 1 | 0 | 4 |
| Xenopus: | | | | | |
| Xenopus Adprhl1 | 35 | 0 | 0 | 2 | 33 |
| (FLAG)Xenopus Adprhl1 | 43 | 4 | 20 | 4 | 15 |
| Hybrid proteins, N-terminal switch: | | | | | |
| (FLAG)human1-94-Xenopus95-354 Adprhl1 | 14 | 6 | 5 | 1 | 2 |
| (FLAG)Xenopus1-94-human95-354 Adprhl1 | 12 | 7 | 5 | 0 | 0 |
| Hybrid proteins, C-terminal switch: | | | | | |
| (FLAG)human1-265-Xenopus266-354 Adprhl1 | 18 | 12 | 3 | 1 | 2 |
| (FLAG)Xenopus1-265-human266-354 Adprhl1 | 20 | 4 | 4 | 7 | 5 |
| Hybrid proteins, N-terminal, small peptide switches: | | | | | |
| Human1-52-Xenopus53-354 Adprhl1 | 27 | 3 | 4 | 3 | 17 |
| Xenopus1-52-human53-94-Xenopus95-354 Adprhl1 | 18 | 0 | 1 | 2 | 15 |
| Human1-19-Xenopus20-31-human32-52-Xenopus53-354 Adprhl1 | 23 | 3 | 6 | 2 | 12 |
| Xenopus1-19-human20-31-Xenopus32-354 Adprhl1 | 22 | 0 | 2 | 3 | 17 |
| Silent adprhl1 cDNA nucleotide changes: | | | | | |
| Xenopus Adprhl1(silent 1-156bp) (36 nucleotides) | 22 | 3 | 4 | 2 | 13 |
| Xenopus Adprhl1(silent 1-282bp) (69 nucleotides) | 21 | 2 | 4 | 2 | 13 |
